# Population Genetic Considerations Regarding Evidence for Biased Mutation Rates in *Arabidopsis thaliana*

**DOI:** 10.1093/molbev/msac275

**Published:** 2022-12-27

**Authors:** Brian Charlesworth, Jeffrey D Jensen

**Affiliations:** Institute of Evolutionary Biology, School of Biological Sciences, University of Edinburgh, Edinburgh EH8 9PU, United Kingdom; School of Life Sciences, Arizona State University, Tempe, 85281 AZ

**Keywords:** population genetics, mutation rates, purifying selection, Tajima's D

## Abstract

It has recently been proposed that lower mutation rates in gene bodies compared with upstream and downstream sequences in *Arabidopsis thaliana* are the result of an “adaptive” modification of the rate of beneficial and deleterious mutations in these functional regions. This claim was based both on analyses of mutation accumulation lines and on population genomics data. Here, we show that several questionable assumptions were used in the population genomics analyses. In particular, we demonstrate that the difference between gene bodies and less selectively constrained sequences in the magnitude of Tajima's *D* can in principle be explained by the presence of sites subject to purifying selection and does not require lower mutation rates in regions experiencing selective constraints.

Monroe et al. (2022) have made the intriguing claim that functionally significant regions of the genome in *Arabidopsis thaliana*, notably gene bodies, have lower mutation rates than genomic regions that are subject to lower levels of selective constraints. This claim was based on analyses of sequence data from mutation accumulation lines, as well as population genomics data from natural populations. Their analysis of the mutation experiments has recently been criticized by Liu and Zhang (2022). Hence, our purpose here is not to further evaluate the experimental evidence on whether de novo mutations are less likely to occur in gene bodies than in flanking sequences but rather to question whether such an effect can reliably be inferred from the population genomic data.

It is first important to note that Monroe et al. (2022) made several inaccurate statements about the population genetics of selection and mutation. For example, they asserted that “the common observation that genetic variants are found less often in functionally constrained regions of the genome is believed to be due solely to selection after random mutation.” In fact, several standard population genetics methods developed to test for selection on DNA sequence variants, such as the HKA test (Hudson et al. 1987), were designed to take into account possible differences in mutation rates among different genes or genomic regions. The common use of comparisons of synonymous versus nonsynonymous polymorphism or divergence levels as indicators of purifying selection on nonsynonymous mutations, described in standard texts such as Graur and Li (2000) and Charlesworth and Charlesworth (2010), also largely corrects for possible mutation rate differences between different genomic regions, by comparing nucleotides from the same coding sequences.

Second, variation in mutation rates across the genome has been documented many times (e.g., Smith et al. 2018). In some cases, highly localized differences in mutation rates have been found to correlate with polymorphism and divergence levels (Reijns et al. 2015). Such variation does not challenge standard evolutionary theory; population genetics theory incorporates mutation rates as well as the strength of selection when modeling levels of variability within populations and divergence between populations over time (see Charlesworth and Charlesworth 2010).

It is important to note that mutation rate heterogeneity does not imply that mutations of a certain type are more likely to arise when they are favored by selection. This possibility has commonly been raised as a challenge to neo-Darwinism; such claims have repeatedly been found to be inconsistent with the data (Charlesworth et al. 2017). Although Monroe et al. (2022) do not appeal to directed mutation of this kind, they compared their concept of mutational bias to mutations behaving as rolls of loaded dice, in which there is a reduced probability of rolling deleterious mutations and an increased probability of rolling beneficial mutations. It is unclear, however, how their postulated mutational bias could increase the frequency of beneficial mutations relative to that of deleterious mutations.

Indeed, the evolution of reduced mutation rates in gene bodies would simply be a special case of the well-studied process of selection for lower mutation rates in response to mutational load, which explains the existence of elaborate error-correction mechanisms that greatly reduce the genome-wide mutation rate (Drake et al. 1998). Modeling shows, however, that selection coefficients on modifiers of mutation rates are very small when mutation rates have reached the levels characteristic of contemporary species (Drake et al. 1998; Lynch et al. 2016). This finding alone casts doubt on the “adaptive mutation bias” suggested by Monroe et al. (2022)—and thus also on its significance in “challenging a long-standing paradigm regarding the randomness of mutation”—but it does not definitively rule it out.

## The Effects of Purifying Selection and Mutation Rates on Estimates of Tajima's *D*

The major argument from the population genomic data presented by Monroe et al. (2022) in favor of lower mutation rates in gene bodies comes from estimates of Tajima's *D* statistic (Tajima 1989b). They estimated *D* over 100 basepair windows for 1,135 genomes sampled from world-wide natural populations of *A. thaliana* and found lower mean *D* values in gene bodies than in upstream and downstream regions. In attempting to distinguish between variation in the mutation rate versus variation in the strength of selection as the underlying explanation of observed levels and patterns of variation, they stated that “reduced mutation rates lead to less negative *D*, whereas purifying selection leads to more negative *D*.” They then asserted that “if *D* is more negative in regions with lower polymorphism, this could indicate that purifying selection is the dominant force underlying lower rates of variation. By contrast, if *D* is less negative in regions of low polymorphism, this would indicate that lower mutation rate is the primary force responsible for lower rates of variation.”

There are several problems with these statements and interpretations. First, Monroe et al. (2022) stated that “Theory shows that … lower mutation rate causes a depletion of rare alleles (fewer young alleles),” that is it causes an apparent skew away from rare variants in the site frequency spectrum (SFS) of segregating variants, relative to the equilibrium neutral expectation. Tajima's equations (Tajima 1989b) were, however, based on the infinite sites mutational model (Kimura 1971), under which the product of the effective population size and the mutation rate is so small that a given polymorphic site segregates for only an ancestral and a single derived variant, and the SFS is independent of the mutation rate. This model is probably very accurate for *A. thaliana*, whose sequence variability is very low within local populations (Nordborg et al. 2005), so that this statement of Monroe et al. (2022) cannot be correct.

Nevertheless, it is true that the expected value of *D* is affected by a sequence's net mutation rate (the product of its number of bases and the mutation rate per nucleotide site; Tajima 1989b), even for neutral variation in a population at equilibrium with respect to mutation and genetic drift. This is true even under the infinite sites model, where there is by definition no skew toward low frequency variants. This is because the *D* statistic involves the ratio of a measure of the skew in the SFS to its standard deviation for a population at neutral equilibrium. The numerator of *D* is equal to the difference between the number of differences between a pair of sequences (*k*),] and the number of segregating sites in the sample (*S*) divided by Watterson's correction factor *a*_1_ (see Equation [S1a] in the Supplementary material online). The function in the denominator that represents the standard deviation of *D* increases more slowly with the number of segregating sites in the sample (*S*) than does the numerator (whose expected value is zero at neutral equilibrium), and the two are correlated in such a way that the expectation of −*D* becomes increasingly large as the expectation of *S* increases. The expectations of both *S* and −*D* are thus increasing functions of the mutation rate, generating a negative correlation between the level of polymorphism and −*D* across loci with different mutation rates. A formal demonstration is provided in the Supplementary material online, section 1 (see Equation S7). Because of this property, *D* is not a good statistic for quantifying the skew in the SFS for population genomic data, and alternative statistics have been proposed for this purpose (Schaeffer 2002; Langley et al. 2014; Becher et al. 2020). The use of this statistic is especially problematic for short sequences, such as those used by Monroe et al. (2022), since *S* must fluctuate greatly between windows, even with a large sample size, and a substantial proportion will have *S* = 0, causing *D* to be set to zero.

These considerations alone do not, however, address the question of why there should be a smaller mean value of −*D* in gene bodies than in adjacent upstream or downstream regions, as seen in figure 2 of Monroe et al. (2022). We show here that this can be explained either by a difference in mutation rates between these genomic regions (as they proposed) or by the fact that gene bodies contain a mixture of nearly neutral and selected sites, notably synonymous and nonsynonymous sites. Importantly, the population genomics data from *A. thaliana* show considerable departures from the assumptions needed for the standard equilibrium model to be applied to neutral or very weakly selected 4-fold degenerate and intergenic sites. For example, figure 1 of Nordborg et al. (2005) shows that mean *θ_w_* (Watterson's measure of diversity per basepair, based on the number of segregating sites; Watterson 1975) for intergenic sites in a sample of 96 genomes is approximately 33% larger than mean *π*, the pairwise diversity per basepair. This implies the existence of a distortion in gene trees toward longer than expected external branches, likely to be caused either by population expansion, hitchhiking, or both (reviewed by Charlesworth and Jensen 2021), as do the strongly negative mean *D* values for upstream and downstream sequences reported by Monroe et al. (2022).

To deal with this problem, we have developed the following simple model, which enables Tajima's *D* statistic to be calculated for a mixture of neutral and selected sites and illuminates the contributions of both selection and the mutation rate to the shape of the SFS for such a mixture. Importantly, the effects of both the mutation rate and selection on *D* are strongly affected by the level of distortion of the SFS in neutral sequences, with large reductions in the magnitude of *D* in sequences containing sites subject to selection being much more marked when there is a high degree of skew of the SFS toward low frequency variants in neutral sequences.

The model assumes that the proportion of effectively neutral sites in the genomic segments under consideration is *p_n_*. Segments that contain only neutral sites (*p_n_* = 1) have a mean pairwise nucleotide site diversity *π_n_* and a mean Watterson's theta per nucleotide site of *θ_wn_*. In general, *π_n_* ≠ *θ_wn_*, with the relationship between them being determined by demography and the effects of selection at linked sites. Selected sites are assumed to be subject to such strong selection that deleterious alleles are present at a mean frequency of $\bar{q}$ per site, where $\bar{q}$ is the infinite population value for equilibrium between mutation and selection. We also assume that $\bar{q}$ << 1/*n*, where *n* is the number of haploid genomes sampled from the population; this implies that the selection coefficients against the deleterious mutations are much greater than the mutation rate per site multiplied by *n*. The probability that a site subject to selection is segregating for a deleterious variant is then approximately *n*$\bar{q}$. The means of *π* and *θ_w_* at the selected sites are approximately 2 $\bar{q}$ and *n*$\bar{q}$ /*a*_1_, respectively.

For the further mathematical development of this model, see section 2 of the Supplementary material online. The formulae derived there enable calculations to be made of the approximate mean values of Tajima's *D* and another measure of the distortion of the SFS toward low frequency variants, Δ*θ*_*w*_ = 1–*π*/*θ*_*w*_ (Becher et al. 2020). A complication in applying these formulae to the results of Monroe et al. (2022) is that their figure 2 shows only the genomic means of *D* and the total numbers of variants estimated from sets of 100 bp windows; the mean numbers of segregating sites (*S*) per 100 basepairs are not presented. It is, however, possible to estimate a mean *S* for a set of 100 basepairs from the mean *D* value for a neutral sequence, given the corresponding mean *π* value, as shown in section 3 of the Supplementary material online. Figure 2 of Monroe et al. (2022) shows that−$\bar{D}$ for upstream and downstream sequences far from gene bodies is approximately 0.9, a very high value that corresponds to a Δ*θ_w_* of approximately 0.54 for the sample size of 1,135 genomes used by Monroe et al. (2022), assuming a *π* value of 0.003 (see supplementary table S1, Supplementary Material online). Given a $\bar{D}$ value for a neutral or nearly neutral part of the genome, application of the corresponding neutral $\bar{S}$ to the equations in part 2 of the Supplementary material yields estimates of $\bar{D}$ and Δ*θ*_*w*_ for the gene bodies, using the model described above. The results are conditional on the assumed proportion of neutral versus strongly selected sites in the gene bodies, and on the assumed strength of selection.

**Fig. 1.**
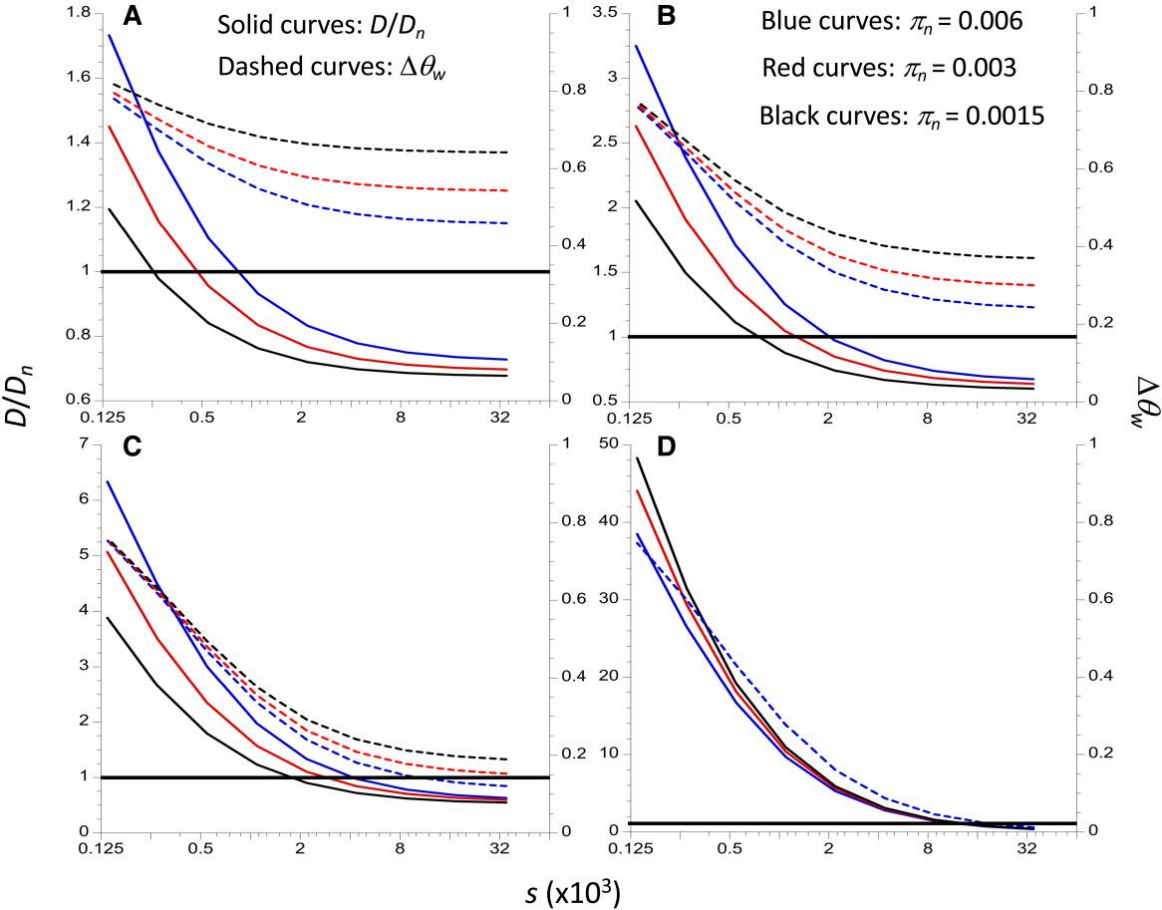
The solid curves display the ratios of the mean *D* for 100 basepair mixed sequences to the mean *D* for 100 basepair neutral sequences, as functions of the selection coefficient against deleterious mutations, *s* (left-hand Y axes). Note that the scale of these axes differs by panel. The dashed curves display the corresponding measure of skew, Δ*θ_w_* (right-hand Y axes). The X-axis uses a log_2_ scale. The proportion of neutral sites in the mixed sequences is 0.45. (*A*–*C*) These panels have mean neutral *D* values of −0.9, −0.45, and −0.225, respectively. (*D*) This panel assumes that there is no distortion of the neutral SFS away from its shape at equilibrium under mutation and drift with constant population size; the mean neutral *D*s are slightly negative in this case. Results for several neutral *π* values (*π_n_*) are shown, corresponding to different mutation rates per basepair (*u* = 7 ×10^–9^ for *π_n_* = 0.003), as indicated by the different colored curves. In panel (*D*), the mutation rate has no effect on Δ*θ_w_*, so that only one curve is shown. In each case, the mutation rates are the same for neutral and mixed sequences.

Figure 1 shows the results for a range of selection coefficients (*s*) against deleterious mutations, using several different values of $\bar{D}$ for purely neutral sequences, including the case when there is no distortion of the gene trees (as shown above, $\bar{D}$ is slightly negative in this case). Three different values of the neutral diversity *π_n_* were used, corresponding to three different mutation rates; *u* for all classes of site was set to 7 ×10^−9^ (Liu and Zhang 2022) for the middle *π_n_* value, 0.003; *π_n_* = 0.006 and 0.0015 correspond to mutation rates 2-fold higher and lower than this, respectively. The proportion of neutral sites in the gene bodies was set to 0.45, consistent with a high proportion of nonsynonymous mutations being subject to strong purifying selection, with the remaining nonsynonymous and silent mutations behaving as neutral or nearly neutral. The selection coefficients were determined on the basis that selection in the highly selfing populations of *A. thaliana* is predominantly against homozygous mutations, so that $\bar{q}=u/s$ for strongly selected variants, where *s* is the selection coefficient against mutant homozygotes; assigned mean frequencies of deleterious mutations were used to determine *s*.

**Fig. 2.**
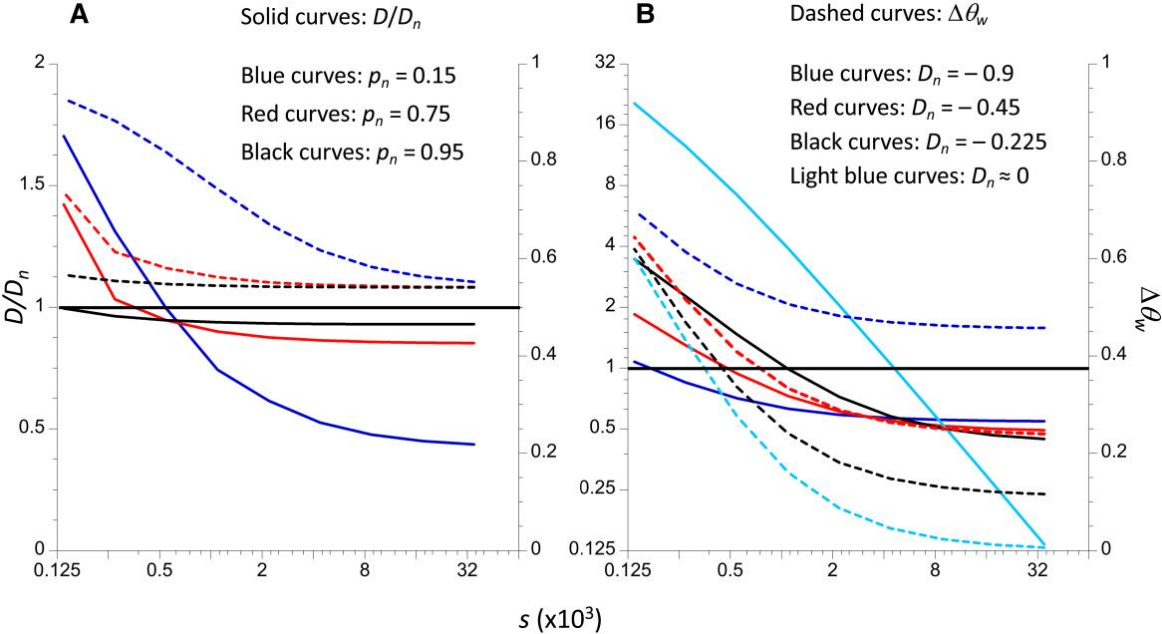
The solid curves display the ratios of the mean *D* for 100 basepair mixed sequences to the mean *D* for 100 basepair neutral sequences, as functions of the selection coefficient against deleterious mutations, *s* (left-hand Y axes). Note that the scale of these axes differs by panel, and a log_2_ scale is used for the left-hand Y axes in order to display the lower values of mean *D* more accurately. The dashed curves display the corresponding measure of skew, Δ*θ_w_* (right-hand Y axes). The X-axis uses a log_2_ scale. (*A*) The different colored curves correspond to different proportions of neutral sites in the mixed sequences; the neutral *π* is 0.003, and the neutral mean *D* is−0.9. The mutation rate (7 × 10^−9^) is the same for both neutral and functional sites. In contrast, in panel (*B*) the mutation rate is 1.4 × 10^−8^ for purely neutral sequences and 7 × 10^−9^ for mixed sequences, with *π* = 0.006 for purely neutral sequences. The different colored curves correspond to different mean *D* values for neutral sequences (the light blue curves correspond to the case of no skew in the neutral SFS).

It can be seen that, with sufficiently strong selection, $-\bar{D}$ with a mixture of neutral and selected sites is less than $-\bar{D}\;$ for neutral sites, that is the ratio of the two values is less than 1, reflecting the fact that there are far fewer polymorphisms in the sample at selected sites. The ratio decreases with the strength of selection, as does Δ*θ*_*w*_. This is despite the fact that segregating selected sites have only singleton variants, which is the maximum possible skew toward low frequencies in the SFS. Importantly, these results show that, in principle, there is a wide range of selection coefficients for which the magnitude of $\bar{D}$ is substantially smaller in sequences with a mixture of neutral and strongly selected sites than in purely neutral sequences with the same mutation rate, the pattern observed by Monroe et al. (2022). These results also demonstrate that, for the same strength of selection, the magnitude of $\bar{D}$ increases with the mutation rate, but here there is no difference in mutation rate among neutral and mixed sequences. In contrast, Δ*θ_w_* for mixed sites decreases with the mutation rate, except when there is no skew in the neutral SFS, at which point it is independent of the mutation rate (panel D). The values of Δ*θ_w_* for the largest selection coefficients in figure 1 are approximately the same as the corresponding values for neutral sites (see supplementary table S1, Supplementary Material online), reflecting the fact that very strongly selected mutations do not segregate in samples of the size used here.

Some insight into the reason for the behavior of $\bar{D}$ as a function of the proportion of neutral sites and the strength of selection can be obtained as follows. Here, the numerical results (supplementary table S1, Supplementary Material online) show that, unless $\bar{D}$ for neutral sequences is close to 0, the main determinant of $\bar{D}$ is the “theoretical value” obtained by substituting $\bar{k}$ and $\bar{S}$ into Equation (S1a), so that:

(1a) $$\bar{D}\approx \;\frac{\bar{k}-\bar{S}a_{1}^{-1}}{\sqrt{(e_{1}-e_{2})\bar{S}+e_{2}{\bar{S}}^{2}}}$$

With sufficiently strong selection, deleterious mutations contribute very little to the values of $\bar{k}$ and $\bar{S}$ for mixed sequences, so that $\bar{k}\approx p_{n}{\bar{k}}_{n}$ and $\;\bar{S}\approx p_{n}{\bar{S}}_{n}$; if there is a skew toward low frequency variants at neutral sites, ${\bar{k}}_{n}<{\bar{S}}_{n}a_{1}^{-1}$. Furthermore, for large sample sizes *e*_2_ << *e*_1_, and the denominator is approximated by $\sqrt{e_{1}p_{n}{\bar{S}}_{n}}$, yielding:

(1b) $$\;\bar{D}\approx \;\frac{\bar{k}-\bar{S}a_{1}^{-1}}{\sqrt{e_{1}\bar{S}}}\propto \sqrt{\;p_{n}}({\bar{k}}_{n}-{\bar{S}}_{n}a_{1}^{-1})$$

It follows that $\bar{D}<0$, and its magnitude increases with *p_n_*, with a maximum when all sites are neutral. However, if selection is sufficiently weak, deleterious mutations contribute to both $\bar{k}\;$ and $\bar{S}$, and the skew toward low frequency variants is higher at the sites under selection, so that this result breaks down. If neutral sites are close to equilibrium, Equation (1) is invalid, again leading to failure of this argument. A large value of $-\bar{D}$ for neutral sites, as in figure 2 of Monroe et al. (2022), is the most favorable situation for detecting an effect of the proportion of neutral sites and the strength of selection on $\bar{D}$, consistent with the results shown in figures 1 and 2. Equation (S11) in section 2 of the Supplementary material online shows that $-\bar{D}$ for neutral sites increases with the mutation rate; by the above argument, this implies that mixed sequences with sites under strong selection also have higher values of $-\bar{D}$ with higher mutation rates, consistent with the results in figure 1. In addition, it can be shown that, for a given value of $\bar{D}$ for neutral sites, the mean number of neutral segregating sites, $\bar{S}$, increases more slowly with an increase in mutation rate than does the mean pairwise number of differences, $\bar{k}$ (see the last paragraph of section 3 of the Supplementary material online). Again, with sufficiently strong selection, this will also apply to the mixed sites. Since Δ*θ_w_* = 1 − $\bar{k}$ /$\bar{S}$, this means that an increase in the mutation rate for a given value of $\bar{D}$ at neutral sites is associated with a reduction in Δ*θ_w_* for mixed sites, provided that selection is sufficiently strong (as seen in fig. 1).

## Difficulties With Population Genetic Inferences in *A. Thaliana*

The large values of −*D* in figure 2 of Monroe et al. (2022) raise some questions that were not discussed by the authors. The value of the scaled mutation rate *M* for a sequence of 100 nucleotides in *A. thaliana* used in their data analyses and simulations is approximately 0.3. Substituting this into Equation (S6a) with their sample size of *n* = 1135, we have −$\bar{D}\approx$ 0.039 for a neutral equilibrium model, which is far smaller in magnitude than the values of 0.8–1.0 in their figure 2 and Extended Data figure 6. There are several possible causes of these large values of −*D*. One is the action of purifying selection on the variants in question, as discussed above, but this seems unlikely to apply to upstream and downstream sequences that are probably under relatively weak selective constraints (Monroe et al. 2022). In addition, a rapid recent population expansion, or several other demographic processes, can cause an increased skew toward rare neutral variants (Tajima 1989a), although this could be offset by the high degree of population structure observed in *A. thaliana* (Sharbel et al. 2000; Nordborg et al. 2005), which causes an excess of intermediate frequency variants when individuals are sampled from multiple populations (Wakeley and Aliacar 2001).

The very low effective recombination rate in *A. thaliana*, caused by the high level of homozygosity associated with its high frequency of self-fertilization (Nordborg et al. 2005; Monroe et al. 2022), is likely to result in a strong effect of background selection caused by linked deleterious mutations (Charlesworth et al. 1993; Barrett et al. 2014). This can distort gene genealogies toward longer external branches, giving a higher frequency of rare neutral variants than in the absence of selection, as is seen in simulations of self-fertilizing populations (Charlesworth et al. 1993). Indeed, simulation results with constant population sizes, presented in the Extended Data Figure of Monroe et al. (2022), showed large values of −*D* both inside and outside gene bodies; these must reflect the effects of background selection. With the very low effective rate of recombination in *A. thaliana*, these effects would be expected to extend over considerable genomic distances and so are unlikely to be the sole explanation for the differences between gene bodies and flanking sequences seen in their figure 2. In addition, *A. thaliana* has had a very complex demographic history involving post-glacial spread from several refugia, as well as experiencing a high degree of population structure (Sharbel et al. 2000; Fulgione et al. 2017). This must cause the site frequency spectra at neutral sites in the world-wide samples used by Monroe et al. (2022) to differ greatly from expectations based on simplified population genetic assumptions.

In summary, the nontrivial interactions between population history, population structure, and the effects of purifying and background selection in *A. thaliana*, particularly as modulated by its low effective recombination rate, require more careful modeling before claims for important effects of mutation heterogeneity on patterns of variability can be accepted. The simple model that we present here does not, of course, deal with these complexities, which present formidable (and possibly insuperable) problems for inference (Johri et al. 2022). It suffices, however, to show that the magnitude of Tajima's *D* can be affected by the presence of sites under purifying selection in a way that is consistent with the patterns reported by Monroe et al. (2022), without requiring any mutation rate differences. Thus, while their “adaptive mutation bias” is valid as a hypothesis, our results demonstrate that it is premature as a conclusion.

## Supplementary Material

msac275_Supplementary_Data

## Data Availability

No new data were generated. Computer output and code are available in the Supplementary material.

## References

[msac275-B1] Barrett SCH , Arunkumar R, Wright SI. 2014. The demography and population genomics of evolutionary transitions to self-fertilization in plants. Phil Trans R Soc B. 369:20130344. 10.1098/rstb.2013.0344 PMC4071518 24958918

[msac275-B2] Becher H , Jackson BC, Charlesworth B. 2020. Patterns of genetic variability in genomic regions with low rates of recombination. Curr Biol. 30:94–100. 31866366 10.1016/j.cub.2019.10.047

[msac275-B3] Charlesworth B , Charlesworth D. 2010. Elements of evolutionary genetics. Greenwood Village (CO): Roberts and Company.

[msac275-B4] Charlesworth B , Jensen JD. 2021. Effects of selection at linked sites on patterns of genetic variability. Ann Rev Ecol Evol Syst. 52:177–197. 10.1146/annurev-ecolsys-010621-044528 PMC10120885 37089401

[msac275-B5] Charlesworth B , Morgan MT, Charlesworth D. 1993. The effect of deleterious mutations on neutral molecular variation. Genetics 134:1289–1303. 8375663 10.1093/genetics/134.4.1289 PMC1205596

[msac275-B6] Charlesworth D , Charlesworth B, Barton NH. 2017. The sources of adaptive variation. Proc R Soc B. 284:20162864. 10.1098/rspb.2016.2864 PMC5454256 28566483

[msac275-B7] Drake JW , Charlesworth B, Charlesworth D, Crow JF. 1998. Rates of spontaneous mutation. Genetics 148:1667–1686. 9560386 10.1093/genetics/148.4.1667 PMC1460098

[msac275-B8] Fulgione A , Koornneef M, Roux F, Hermisson J, Hancock AM. 2017. Madeiran *Arabidopsis thaliana* reveals ancient long-range colonization and clarifies demography in Eurasia. Mol Biol Evol. 35:564–574. 10.1093/molbev/msx300 PMC5850838 29216397

[msac275-B9] Graur D , Li W-H. 2000. Fundamentals of molecular evolution. 4th ed. Sunderland (MA): Sinauer.

[msac275-B10] Hudson RR , Kreitman M, Aguadé M. 1987. A test of molecular evolution based on nucleotide data. Genetics 116:153–159. 3110004 10.1093/genetics/116.1.153 PMC1203113

[msac275-B11] Johri P , Aquadro CF, Beaumont M, Charlesworth B, Excoffier L, Eyre-Walker A, Keightley PD, Lynch M, McVean G, Payseur BA, et al 2022. Recommendations for improving statistical inference in population genomics. PLoS Biol. 20:e3001669. 10.1371/journal.pbio.3001669 PMC9154105 35639797

[msac275-B12] Kimura M . 1971. Theoretical foundations of population genetics at the molecular level. Theor Pop Biol. 2:174–208. 5162686 10.1016/0040-5809(71)90014-1

[msac275-B13] Langley SA , Karpen GH, Langley CH. 2014. Nucleosomes shape DNA polymorphism and divergence. PLoS Genet. 10:e1004457. 10.1371/journal.pgen.1004457 PMC4081404 24991813

[msac275-B14] Liu H , Zhang J. 2022. Is the mutation rate lower in genomic regions of stronger selective constraints? Mol Biol Evol. 39:msac169. 10.1093/molbev/msac169 PMC9372563 35907247

[msac275-B15] Lynch M , Ackerman MS, Gout JF, Long H, Sung W, Thomas WK, Foster PL. 2016. Genetic drift, selection and the evolution of the mutation rate. Nat Rev Genet. 17:704–714. 27739533 10.1038/nrg.2016.104

[msac275-B16] Monroe JG , Srikant T, Carbonell-Bejerano P, Becker C, Lensink M, Exposito-Alonso M, Klein M, Hildebrandt J, Neumann M, Kliebenstein D, et al 2022. Mutation bias reflects natural selection in *Arabidopsis thaliana*. Nature 602:101–105. 35022609 10.1038/s41586-021-04269-6 PMC8810380

[msac275-B17] Nordborg M , Hu TT, Ishino Y, Jhaveri J, Toomajian C, Zheng T, Bakker E, Calabrese P, Gladstone J, Goyal R, et al 2005. The pattern of polymorphism in *Arabidopsis thaliana*. PLoS Biol 3:1289–1299. 10.1371/journal.pbio.0030196 PMC1135296 15907155

[msac275-B18] Reijns MAM , Kemp H, Ding J, Marion de Procé S, Jackson AP, Taylor MS. 2015. Lagging- strand replication shapes the mutational landscape of the genome. Nature 518:502–506. 25624100 10.1038/nature14183 PMC4374164

[msac275-B19] Schaeffer SW . 2002. Molecular population genetics of sequence length diversity in the ADH region of *Drosophila pseudoobscura*. Genet Res. 80:163–175. 12688655 10.1017/s0016672302005955

[msac275-B20] Sharbel TF , Haubold B, Mitchell-Olds T. 2000. Genetic isolation by distance in *Arabidopsis thaliana*: biogeography and post-glacial colonization of Europe. Mol Ecol. 9:2109–2118. 11123622 10.1046/j.1365-294x.2000.01122.x

[msac275-B21] Smith TCA , Arndt P, Eyre-Walker A. 2018. Large-scale variation in the rate of germ-line de novo mutation, base composition, divergence and diversity. PLoS Genet. 14:e1007254. 10.1371/journal.pgen.1007254 PMC5891062 29590096

[msac275-B22] Tajima F . 1989a. The effect of change in population size on DNA polymorphism. Genetics 123:597–601. 2599369 10.1093/genetics/123.3.597 PMC1203832

[msac275-B23] Tajima F . 1989b. Statistical method for testing the neutral mutation hypothesis. Genetics 123:585–595. 2513255 10.1093/genetics/123.3.585 PMC1203831

[msac275-B24] Wakeley J , Aliacar N. 2001. Gene genealogies in a metapopulation. Genetics 159:893–905. 11606561 10.1093/genetics/159.2.893 PMC1461837

[msac275-B25] Watterson GA . 1975. On the number of segregating sites in genetical models without recombination. Theor Pop Biol. 7:256–276. 1145509 10.1016/0040-5809(75)90020-9

