# Correction to: Population Genetic Considerations Regarding Evidence for Biased Mutation Rates in Arabidopsis thaliana

**DOI:** 10.1093/molbev/msad065

**Published:** 2023-03-24

This is a correction to: Brian Charlesworth, Jeffrey D Jensen, Population Genetic Considerations Regarding Evidence for Biased Mutation Rates in *Arabidopsis thaliana, Molecular Biology and Evolution*, Volume 40, Issue 2, February 2023, msac275, https://doi.org/10.1093/molbev/msac275

In the originally published version of this manuscript, there were typos in Equations (S6a) and (S6b) of the Supplementary Information. A new version of the Supplementary Information has been added, which includes the corrected equations.

