# Supplementary material for: Population Genetic Considerations Regarding Evidence for Biased Mutation Rates in Arabidopsis thaliana
Source: Mol Biol Evol. 2022 Dec 27;40(2):msac275. doi: 10.1093/molbev/msac275 (PMC9907473; doi:10.1093/molbev/msac275)
Supplement: msac275_Supplementary_Data [file msac275_supplementary_data.pdf]

Supplementary Information: Derivations of the Equations in the Main Text

1. Properties of Tajima's *D* under the equilibrium infinite sites neutral model

Tajima's *D* statistic for a defined portion of the genome is defined as follows (Equation 38 of Tajima 1989):

$$D = \frac{d}{\sqrt{(e_1 - e_2)S + e_2 S^2}} \qquad \text{(S1a)}$$

where S is the observed number of segregating sites in a sample of n genomes, k is the mean pairwise differences between sequences, and d is defined as:

$$d = k - S/a_1 \qquad \text{(S1b)}$$

For large n, as in Monroe et al. (2022), the constants a_1, a_2, e_1 and e_2 (defined by Tajima's Equations 3, 4, 36 and 37) can be approximated as follows (~ denotes values for large n):

$$a_1 \sim \ln(n) + 0.5772 \qquad \text{(S2a)}$$

$$a_2 \sim \frac{\pi^2}{6} \approx 1.6449 \qquad \text{(S2b)}$$

$$e_1 \sim \left(\frac{1}{3} - \frac{1}{a_1}\right)\frac{1}{a_1} \qquad \text{(S2c)}$$

$$e_2 \sim \left(\frac{2}{9} - \frac{1}{a_1}\right)\frac{1}{a_1^2} \qquad \text{(S2d)}$$

For further work, we write:

$$D = d\, f(S)^{-\frac{1}{2}} = d\, g(S) \qquad \text{(S3a)}$$

where

$$f(S) = (e_1 - e_2)S + e_2 S^2 \qquad \text{(S3b)}$$

At equilibrium under drift and neutral mutation in a single population of constant effective size, the expectations of k and S/a_1 are both equal to M, the scaled mutation rate for a whole sequence. $M = 4N_e mu$, where N_e is the effective population size, m is the number of basepairs in the sequence, and u is the mutation rate per basepair (Watterson 1975). Note that we use θ to denote the scaled mutation rate per basepair, so that $M = m\theta$. Equation (S3) implies that in this case E{d} = 0.

Using overbars to indicate expectations, we can write $d = \bar{d} + \delta d, g(S) = g(\bar{S}) + \delta g$, where the expectation of δd is zero for the case of equilibrium under neutrality. In general, $\mathrm{E}\{D\} = \bar{d}g(\bar{S}) + \mathrm{E}\{(\delta d)\}g(\bar{S}) + \bar{d}\mathrm{E}\{(\delta g)\} + \mathrm{E}\{(\delta d)(\delta g)\}$. In the present case, $\bar{d} = 0$, so that this expression reduces to $\mathrm{E}\{D\} = \mathrm{E}\{(\delta d)(\delta g)\}$.

Using the first term in the Taylor series expansion of $g(S)$ around $\bar{S}$, the approximate expectation of D, to the accuracy of second-order terms in δd and $S - \bar{S}$, is given by:

$$\bar{D} = \mathrm{E}\{(\delta d)(\delta g)\}$$

$$\approx -\mathrm{E}\{(\delta d)\ (S - \bar{S})\} \left(\frac{\partial f}{\partial s}\right)_{\bar{S}} \frac{1}{2} f(\bar{S})^{-\frac{3}{2}}$$

$$= \frac{1}{2}\{[Var(S)/a_1] - Cov(k, S)\} \left(\frac{\partial f}{\partial s}\right)_{\bar{S}} f(\bar{S})^{-\frac{3}{2}} \qquad \text{(S4)}$$

Using Tajima's Equations (1), (2) and (25), for large we have:

$$\bar{S} = a_1 M \qquad \text{(S5a)}$$

$$Cov(k, S) \approx M + \tfrac{1}{2}M^2 \qquad \text{(S5b)}$$

$$Var(S) = a_1 M + a_2 M^2 \qquad \text{(S5c)}$$

$$f(\bar{S}) = (e_1 - e_2)\bar{S} + e_2 \bar{S}^2 \qquad \text{(S5d)}$$

$$\left(\frac{\partial f}{\partial s}\right)_{\bar{S}} = (e_1 - e_2 + 2e_2\bar{S}) \qquad \text{(S5e)}$$

Substituting these expressions into Equation (S4) and simplifying, we obtain:

$$\bar{D} \approx -\frac{M^{\frac{1}{2}}}{4a_1^{2.5}}(a_1 - 2a_2)\ [(e_1 - e_2) + e_2 a_1\ M]^{-\frac{3}{2}}\ [(e_1 - e_2) + 2e_2 a_1 M] \qquad \text{(S6a)}$$

A sufficient condition for $\bar{D} < 0$ from these expressions is $e_1 - e_2 > 0$ and $a_1 > 2a_2$. Using Equations (S2c) and (S2d), it is easily seen that this requires a_1 to exceed the smaller root of the quadratic equation $a_1^2 - \frac{11}{3}a_1 + 3 = 0$, i.e., $a_1 > 1.233$. Since the minimal sample size for a meaningful estimate of D is 3, corresponding to $a_1 = 1.5$, this condition is automatically satisfied. From Equations (S2a) and (S2b), $a_1 > 2a_2$ is satisfied if $n > 15$.

For $a_1 >> a_2$, Equation (S6a) can be approximated by:

$$\bar{D} \approx -(\frac{3M}{16})^{\frac{1}{2}}\ a_1^{-2}\left[1 + \tfrac{2}{3}M\right]^{-\frac{3}{2}}\left[\ 1 + \tfrac{4}{3}M\right] \qquad \text{(S6b)}$$

Furthermore, Equation (S6a) implies that:

$$\frac{d\bar{D}}{dM} \propto -\ (e_1 - e_2) + 3e_2 a_1 M\{2 - \frac{[(e_1 - e_2) + 2e_2 a_1 M]}{[(e_1 - e_2) + e_2 a_1 M]}\} \qquad \text{(S7)}$$

When $e_1 - e_2 > 0$, the second term inside the braces in Equation (S7) is ≤ 2, so that the whole right-hand side of Equation (S7) is always negative under this condition, implying that the magnitude of $\overline{D}$ is an increasing function of M.

2. Evaluating Tajima's *D* with a mixture of neutral and selected sites

Here, we no longer assume that neutral sites are at mutation-drift equilibrium, and analyze the model of a mixture of neutral and selected sites described in the main text. We first examine the theoretical value of Tajima's D, D^*, defined here as the value of D obtained by substituting the mean values of the relevant parameters into Equation (S1a). Let the number of nucleotide sites in each of the segments of the genome under consideration be m. The expected sum of the diversities across these sites is thus $\bar{k} = m[p_n\pi + 2(1-p_n)\,\bar{q}]$, where $\bar{q}$ is the mean frequency of deleterious mutations at the selected sites. The expected total number of segregating sites is $\bar{S} = m[p_n\theta_w a_1 + (1-p_n)n\bar{q}]$, where is Watterson's theta statistic (Watterson 1975) and n is the sample size. D^* is given by:

$$D^* = \frac{\bar{d}}{\sqrt{(e_1 - e_2)\bar{S} + e_2\bar{S}^2}} \qquad \text{(S8a)}$$

where $\underline{d}$ is obtained from the equivalent of Equation (S1b):

$$\bar{d} = \bar{k} - \bar{S}/a_1 \qquad \text{(S8b)}$$

As before, the constants a_1, e_1 and e_2 are defined by Tajima's Equations (3), (4), (36) and (37) (see Equations (S2) for their large-sample values).

If the diversities and numbers of segregating sites are estimated for many independent segments of the genome, their means over the segments in question can be used to approximate the theoretical values of π and θ_w. However, the result in Monroe *et al.* (2022) were obtained by taking the mean of D across large numbers of small segments; the denominator in Equation (S8a) is thus highly variable, which needs to be considered when estimating the expected value of D, as was done above for the equilibrium neutral case. We first examine the case where there are only neutral sites ($p_n = 1$). As before, we can write:

$$D_n = d_n f(S_n)^{-1/2} = d_n g(S_n) \qquad \text{(S9a)}$$

where

$$f(S_n) = (e_1 - e_2)S_n + e_2 S_n^2, \;\; g(S_n) = f(S_n)^{-0.5} \qquad \text{(S9b)}$$

The approximate expectation of D_n is now given by:

$$\bar{D}_n \approx \bar{d}_n g(\bar{S}_n) - \{Cov(k_n, S_n) - Var(S_n)/a_1\}\left(\frac{\partial g}{\partial S_n}\right)_{\bar{S}_n} + \frac{1}{2}\bar{d}_n Var(S_n)\left(\frac{\partial^2 g}{\partial S_n^2}\right)_{\bar{S}_n} \qquad \text{(S10)}$$

where $\bar{d}$ is no longer zero as was assumed earlier. Expressions for the derivatives in this equation are obtained by setting $p_n = 1$ in Equations (S13) below.

If the population is not at mutation-drift equilibrium, it is difficult to evaluate the covariances and variances in Equation (S10). However, approximations for these terms can be found as follows. As shown in section 4 below, the covariance between k_n and S_n tends to M as the gene genealogy approaches a star phylogeny, in which the n branches of the gene tree accumulate mutations independently of each other, so that Equation (S5b) is likely to be the upper bound to the covariance for a given value of M_n if neutral sites are skewed towards low frequency variants, as is the case in *A. thaliana* (Monroe et al. 2022). In addition, Equation (S5c) shows that, unless $M_n >> 1/a_1$, $var(S_n)/a_1$ for the equilibrium case is close to the ratio of the mean of S_n to a_1. Similarly, for a star phylogeny, $var(S_n)/a_1 = \bar{S}_n/a_1$ (see section 4). This implies that $\bar{S}_n/a_1$ should also provide a good approximation to $var(S_n)/a_1$ for cases with distortions of the gene tree shapes towards longer external branches. The empirical values of π, θ_w and S for putatively neutral sites obtained from averaging over large number of segments can then be substituted into the expressions for d_n, D_n and the derivative of $g(S_n)$ in Equation (S10), in order to obtain approximations for the expectation of D_n. Conversely, given estimates of π and $\overline{D}_n$, an approximate estimate of the expectation of S_n can be obtained, as shown in section 3 below.

It is useful to note that numerical examples suggest that the theoretical value of $\overline{D}_n$, $D^* = \bar{d}g(\bar{S}_n)$, is usually the dominant term in Equation (S10), even when there is a distortion of the gene trees towards longer external branches, so that $\bar{d}g(\bar{S}_n) < 0$ (see the "Approx. correction term for mean D" in the sections of Supplementary Table S1 headed "Mean frequency of deleterious alleles").

Since both the expected pairwise diversities and numbers of segregating sites are proportional to the mutation rate under the infinite sites model, for a non-equilibrium population we can write $\bar{k}_n = A_n M$ and $\bar{S}_n = B_n M$, where A_n and B_n depend only on the

properties of the gene trees; $A_n - B_n/a_1 < 0$ when $D_n^* < 0$. This yields the following expression:

$$D_n^* = \left(A_n - \frac{B_n}{a_1}\right)\{[(e_1 - e_2)M_n^{-1} + e_2 B_n]B_n\}^{-\frac{1}{2}} \tag{S11}$$

It is evident from this expression that the magnitude of D_n^* increases with M_n when $D_n^* < 0$, generalizing the result derived for the case of equilibrium in section 1.

The properties of sites under strong selection (denoted by the subscript s) can be evaluated as follows. Under the assumptions described in the main text, the probability of detecting a segregating mutation at an individual site in a sample of size n is $n\bar{q}$, with at most one copy of a mutation at a given site. The number of such mutations on a single gene tree is Poisson-distributed (ignoring any variance in q among sites), and is independent of the size of the tree, so that the mean and variance of S_s are both equal to $\bar{S}_s = m_s n\bar{q}$, where $m_s = (1 - p_n)m$. Similarly, the mean of k for the selected sites is $\bar{k}_s = 2m_s\bar{q}$. A straightforward calculation (see section 4) gives $Cov(k_s, S_s) \approx \bar{k}_s$, which can be neglected in comparison to $var(S_s)/a_1$ if n is large.

The expectation of the net value of D for a mixture of neutral and selected sites is given by combining the theoretical value of D (D^*) with the expressions for the variance and covariance terms for the neutral and selected sites. We now write $S = p_n S_n + q_n S_s$, where $q_n = 1 - p_n$. As before, we write:

$$f(S) = S[(e_1 - e_2) + Se_2] \tag{S12}$$

D^* is now the product of $\bar{d} = p_n\left(\bar{k}_n - \frac{\bar{S}_n}{a_1}\right) + q_n\left(\bar{k}_s - \frac{\bar{S}_s}{a_1}\right)$ and $g(\bar{S})$.

The derivatives needed for the additional terms in the equivalent of Equation (S10) are:

$$\left(\frac{\partial g}{\partial S_n}\right)_{\bar{S}} = -\tfrac{1}{2}p_n f(\bar{S})^{-\frac{3}{2}}[(e_1 - e_2) + 2\bar{S}_n e_2] \tag{S13a}$$

$$\left(\frac{\partial g}{\partial S_s}\right)_{\bar{S}} = -\tfrac{1}{2}q_n f(\bar{S})^{-\frac{3}{2}}[(e_1 - e_2) + 2\bar{S}_s e_2] \tag{S13b}$$

$$\left(\frac{\partial^2 g}{\partial S_n^2}\right)_{\bar{S}} = \tfrac{3}{4} p_n^2 f(\bar{S})^{-\frac{5}{2}}[(e_1 - e_2) + 2\bar{S}_n e_2]^2 - p_n e_2 f(\bar{S})^{-\frac{3}{2}} \tag{S13c}$$

$$\left(\frac{\partial^2 g}{\partial S_s^2}\right)_{\bar{S}} = \tfrac{3}{4} q_n^2 f(\bar{S})^{-\frac{5}{2}}[(e_1 - e_2) + 2\bar{S}_s e_2]^2 - q_n e_2 f(\bar{S})^{-\frac{3}{2}} \tag{S13d}$$

$$\left(\frac{\partial^2 g}{\partial S_n S_s}\right)_{\bar{S}} = \tfrac{3}{4}p_n q_n\, f(\bar{S})^{-\frac{5}{2}}[(e_1 - e_2) + 2\bar{S}_n e_2][(e_1 - e_2) + 2\bar{S}_s e_2] \tag{S13e}$$

The correction term involving the first derivatives of g to be added to D^* in order to obtain the full expression for $\bar{D}$ with a mixture of neutral and selected sites is:

$$p_n \left\{Cov(k_n, S_n\,) - \frac{Var(S_n)}{a_1}\right\} \left(\frac{\partial g}{\partial S_n}\right)_{\bar{S}} + q_n \left\{Cov(k_s, S_s\,) - \frac{Var(S_s)}{a_1}\right\} \left(\frac{\partial g}{\partial S_s}\right)_{\bar{S}} \qquad \text{(S14)}$$

The additional correction term to be added to D^* in order to estimate $\bar{D}$ is the sum of the products of the second derivatives in Equations (S13c) - S(13e) with $\bar{d}\ Var(S_n)/2$, $\bar{d}\ Var(S_s)/2$ and $\bar{d}\ Cov(S_n, S_s)$, respectively. This presents the problem that there is no equation for $Cov(S_n, S_s)$. However, this term is likely to be negative in the case of *A. thaliana*, since there is a high level of linkage disequilibrium over the distances considered in the analyses of Monroe et al. (2022), so that selected and neutral variants within the same segment share a gene tree. The presence of deleterious variants must reduce the size of a tree, causing a negative correlation between S_n and S_s. This will offset the contribution from the other two second derivatives, which are positive for the cases under consideration here. Since $\bar{d} < 0$, use of a correction using these derivatives alone is likely to somewhat overestimate the magnitude of $\bar{D}$ for mixed sites and is thus conservative for the purpose of this study. But the magnitude of the mixed second derivative is generally small in comparison with D^* (see Supplementary Table 1), so this correction is likely to be of minor importance.

3. Estimating the mean number of segregating sites from mean *D* for neutral sites

If only a mean D_n value is available, as in Monroe et al. (2022), the following procedure can be used to estimate the mean number of segregating sites for a neutral genomic segment of size m, $\bar{S}_n$. We assume that the mean pairwise diversity statistic $\bar{k}_n$ is accurately estimated, providing an estimate of the scaled mutation rate for neutral segments, M_n. Let the corresponding estimate of mean D be $\bar{D}_n$. An iterative procedure is used, in which initially $\bar{S}_n$ is set to the neutral equilibrium value corresponding to $\bar{k}_n$. The sum of the correction terms involving the first and second derivatives and the variance and covariances, as described above, is denoted here by C (in this case, $p_n = 1$). Writing x for the unknown quantity $\bar{S}_n$, we can write:

$$\bar{D}_n = (\bar{k}_n - x/a_1)[(e_1 - e_2)x + e_2\, x^2]^{-\frac{1}{2}} + C \qquad \text{(S14a)}$$

This can be rearranged to yield a quadratic equation in *x*:

$$ax^2 + bx + c = 0 \qquad \text{(S14b)}$$

with coefficients:

$$a = a_1^{-2} - (\overline{D}_n - C)^2 e_2,\ b = -[(\overline{D}_n - C)^2(e_1 - e_2) + 2\overline{k}_n a_1^{-1}], c = \overline{k}_n^2 \qquad \text{(S14c)}$$

This equation can be solved using the standard formula for a quadratic equation, yielding an estimate of $\bar{S}_n = x$. This estimate replaces the old value of $\bar{S}_n$, and the cycle is repeated until $\bar{S}_n$ stabilizes. In practice, the correction terms are usually small, and there is little change in $\bar{S}_n$ after the first iteration (see the first section of Supplementary Table 1).

It is important to note that the assumption of a fixed value of $\overline{D}_n$ has implications for the behavior of $\bar{S}_n$ as a function of the mutation rate. In the numerical results presented in Figures 1 and 2, different values of the mutation rate lead to different values of $\overline{k}_n$. Inspection of Equation (S11) shows that, for a fixed value of D_n^* (which is close to$\overline{D}_n$ for highly skewed neutral site frequency spectra), the presence of $\bar{S}_n$in the denominator means that the value of $\bar{S}_n$corresponding to a fixed value of D_n^* increases more slowly with the mutation rate than does $\overline{k}_n$, as can be seen in the numerical results in Table S1.

4. Statistical properties of star phylogenies

The results on the covariance between *k* and *S* used in Section 2 are derived in this section. A star phylogeny for a sample of *n* alleles has *n* branches that independently acquire mutations. First consider the neutral case. Under the infinite sites model, a single nucleotide site will only have a mutation on one out of the *n* branches, i.e., there is a singleton variant. Over *m* sites, there can be $S \leq m$ such mutations segregating in the sample, each at a separate site. If the *i*th branch has k_i mutations, we have $S = \sum_i k_i$, where k_i follows a Poisson distribution with mean *M*/2. Because of the independence among branches, *S* follows a Poisson distribution with mean and variance *Mn*/2. For a star phylogeny, a mutation present at a given site on a given branch will not be represented at that site on the other *n* – 1 branches, generating a contribution of (*n* –1) to the pairwise divergence statistic *k*, taken over all *n*(*n* – 1)/2 pairwise comparisons. We thus have:

$$k = \frac{2}{n}\sum_i k_i = \frac{2S}{n} \qquad \text{(S15)}$$

The expectation of k is $\bar{k} = M$ and its variance is $2M/n$, which tends to zero with increasing sample size. From Equation (S15), we have $Cov(k, S) = 2Var(S)/n$, which reduces to $Cov(k, S) = M = \bar{k}$.

A similar argument can be applied to the case of strong selection, where deleterious mutations are present as singletons. In this case, we have $\bar{k}_i = \bar{q}$, so that $\bar{k} = 2m\bar{q}$ and $\bar{S} = nm\bar{q}$. From Equation (S15), $Cov(k, S) = \bar{k}$.

REFERENCES

Monroe JG, Srikant T, Carbonell-Bejerano P, Becker C, Lensink M, Exposito-Alonso M. *et al*. 2022. Mutation bias reflects natural selection in *Arabidopsis thaliana*. *Nature* **602**:101-105.

Tajima F. 1989. Statistical method for testing the neutral mutation hypothesis. *Genetics* **123**:585-595.

Watterson GA. 1975. On the number of segregating sites in genetical models without recombination. Theor. Pop. Biol. 7:256-276.

Supplementary Table S1

Tajima's D with mixture of neutrality and strong selection
Sample size= 1135
Total number of sites in a segment= 100

Proportion of neutral sites in a segment= 0.449999988
Ratio of mutation rates (mixed/pure neutral)= 1.00000000

Observed Tajima D for neutral sites = -0.899999976
Neutral diversity= 3.00000003E-03

Coefficients needed for the Tajima D calculations
a1= 7.61116838 a2= 1.64405346
b1= 0.333921224 b2= 0.222614661
c1= 0.202535346 c2= 0.119377315
e1= 2.66102832E-02 e2= 2.00385135E-03
Equilibrium neutral values of k and S for m neutral sites
kn= 0.300000012 Sn= 2.28335071

Iteration= 1

f(S)= 6.66325763E-02 derivative= 3.37574258E-02
g(S)= 3.87397385 derivative= -0.981317163
2nd derivative of g(S)= 0.629230559
3rd derivative of g(S)= -0.678907394

Iteration= 5

f(S)= 0.171847776 derivative= 4.45298329E-02
g(S)= 2.41228175 derivative= -0.312539697
2nd derivative of g(S)= 9.33509767E-02
3nd derivative of g(S)= -4.58959974E-02

Equilibrium Var(S)= 2.43131542 Cov(S,k)= 0.345079303
1st approx. correction term for mean D= -8.01314879E-03
2nd approx. correction term for mean D= -4.00771014E-02
Coefficients of quadratic for S
a= 1.58079509E-02 b= -9.66896489E-02 c= 9.00000036E-02 discrim= 6.04816116E-02

Estimate of neutral mean S= 4.97127247
Theta-w= 6.53155008E-03 Delta-theta-w= 0.540690899
Check on D= -0.900000036

Uncorrected mean frequency of deleterious alleles= 1.00000001E-07

Mean k for m selected sites (corrected for mutation rate)= 1.99999995E-05
Mean S for m selected sites (corrected for mutation rate)= 1.13500003E-02
Var(Ss)= 1.13500003E-02 Cov(S,k)= 1.99999995E-05
Uncorrected estimate of mean D for selected sites= -8.79949182E-02

Theta-w for selected sites 1.49122970E-05 Delta-theta-w= 0.986588240

Statistics for mixture of m neutral and selected sites
Mean k for mixture=   0.135011002
Mean S for mixture=   2.24331498
d= -0.159728900   f(S)=   6.52842894E-02

1st and 2nd derivs wrt Sn= -0.600648463   0.222486719
1st and 2nd derivs wrt Ss= -0.406415671   6.05378971E-02
Mixed 2nd derivative=   0.187118366
1st approx. correction term for mean D=   -6.60110032E-03
2nd approx. term for mean D=   -4.32563834E-02
Estimate of theoretical D= -0.625142694
Corrected estimate of mean D= -0.675000191

Theta-w=   2.94739893E-03 Delta-theta-w=   0.541931748
Ratio of mixture to neutral D=   0.694602966

Uncorrected mean frequency of deleterious alleles=   2.00000002E-07

Mean k for m selected sites (corrected for mutation rate)=   3.99999990E-05
Mean S for m selected sites (corrected for mutation rate)=   2.27000006E-02
Var(Ss)=   2.27000006E-02 Cov(S,k)=   3.99999990E-05
Uncorrected estimate of mean D for selected sites= -0.124386184

Theta-w for selected sites   2.98245941E-05 Delta-theta-w=   0.986588240

Statistics for mixture of m neutral and selected sites
Mean k for mixture=   0.135021999
Mean S for mixture=   2.24955750
d= -0.160538062   f(S)=   6.54940903E-02

1st and 2nd derivs wrt Sn= -0.597764671   0.220536903
1st and 2nd derivs wrt Ss= -0.405210704   6.03075475E-02
Mixed 2nd derivative=   0.185965955
1st approx. correction term for mean D=   -6.24091690E-03
2nd approx. term for mean D=   -4.31497246E-02
Estimate of theoretical D= -0.627302408
Corrected estimate of mean D= -0.676693022

Theta-w=   2.95560085E-03 Delta-theta-w=   0.543165684
Ratio of mixture to neutral D=   0.697002649

Uncorrected mean frequency of deleterious alleles=   4.00000005E-07

Mean k for m selected sites (corrected for mutation rate)=   7.99999980E-05
Mean S for m selected sites (corrected for mutation rate)=   4.54000011E-02
Var(Ss)=   4.54000011E-02 Cov(S,k)=   7.99999980E-05
Uncorrected estimate of mean D for selected sites= -0.175746575

Theta-w for selected sites   5.96491882E-05 Delta-theta-w=   0.986588240

Statistics for mixture of m neutral and selected sites
Mean k for mixture=   0.135044008
Mean S for mixture=   2.26204252
d= -0.162156433   f(S)=   6.59141764E-02

1st and 2nd derivs wrt Sn= -0.592059255   0.216700271

1st and 2nd derivs wrt Ss= -0.402821541 5.98518774E-02
Mixed 2nd derivative= 0.183691278
1st approx. correction term for mean D= -5.52704977E-03
2nd approx. term for mean D= -4.29377258E-02
Estimate of theoretical D= -0.631603837
Corrected estimate of mean D= -0.680068612

Theta-w= 2.97200447E-03 Delta-theta-w= 0.545613050
Ratio of mixture to neutral D= 0.701781988

Uncorrected mean frequency of deleterious alleles= 8.00000009E-07

Mean k for m selected sites (corrected for mutation rate)= 1.59999996E-04
Mean S for m selected sites (corrected for mutation rate)= 9.08000022E-02
Var(Ss)= 9.08000022E-02 Cov(S,k)= 1.59999996E-04
Uncorrected estimate of mean D for selected sites= -0.248086691

Theta-w for selected sites 1.19298376E-04 Delta-theta-w= 0.986588240

Statistics for mixture of m neutral and selected sites
Mean k for mixture= 0.135088012
Mean S for mixture= 2.28701258
d= -0.165393129 f(S)= 6.67562187E-02

1st and 2nd derivs wrt Sn= -0.580892563 0.209271848
1st and 2nd derivs wrt Ss= -0.398124993 5.89602292E-02
Mixed 2nd derivative= 0.179259583
1st approx. correction term for mean D= -4.12480300E-03
2nd approx. term for mean D= -4.25192192E-02
Estimate of theoretical D= -0.640135050
Corrected estimate of mean D= -0.686779082

Theta-w= 3.00481147E-03 Delta-theta-w= 0.550427675
Ratio of mixture to neutral D= 0.711261153

Uncorrected mean frequency of deleterious alleles= 1.60000002E-06

Mean k for m selected sites (corrected for mutation rate)= 3.19999992E-04
Mean S for m selected sites (corrected for mutation rate)= 0.181600004
Var(Ss)= 0.181600004 Cov(S,k)= 3.19999992E-04
Uncorrected estimate of mean D for selected sites= -0.349566966

Theta-w for selected sites 2.38596753E-04 Delta-theta-w= 0.986588240

Statistics for mixture of m neutral and selected sites
Mean k for mixture= 0.135176003
Mean S for mixture= 2.33695245
d= -0.171866536 f(S)= 6.84477910E-02

1st and 2nd derivs wrt Sn= -0.559492469 0.195336550
1st and 2nd derivs wrt Ss= -0.389046311 5.72520085E-02
Mixed 2nd derivative= 0.170842737
1st approx. correction term for mean D= -1.41821336E-03
2nd approx. term for mean D= -4.17052805E-02
Estimate of theoretical D= -0.656918645
Corrected estimate of mean D= -0.700042129

Theta-w= 3.07042548E-03 Delta-theta-w= 0.559748292
Ratio of mixture to neutral D= 0.729909599

Uncorrected mean frequency of deleterious alleles= 3.20000004E-06

Mean k for m selected sites (corrected for mutation rate)= 6.39999984E-04
Mean S for m selected sites (corrected for mutation rate)= 0.363200009
Var(Ss)= 0.363200009 Cov(S,k)= 6.39999984E-04
Uncorrected estimate of mean D for selected sites= -0.490799010

Theta-w for selected sites 4.77193506E-04 Delta-theta-w= 0.986588240

Statistics for mixture of m neutral and selected sites
Mean k for mixture= 0.135352001
Mean S for mixture= 2.43683243
d= -0.184813365 f(S)= 7.18609318E-02

1st and 2nd derivs wrt Sn= -0.520108700 0.170738414
1st and 2nd derivs wrt Ss= -0.372050315 5.41072153E-02
Mixed 2nd derivative= 0.155619323
1st approx. correction term for mean D= 3.63300089E-03
2nd approx. term for mean D= -4.01757173E-02
Estimate of theoretical D= -0.689424932
Corrected estimate of mean D= -0.725967646

Theta-w= 3.20165372E-03 Delta-theta-w= 0.577243447
Ratio of mixture to neutral D= 0.766027689

Uncorrected mean frequency of deleterious alleles= 6.40000007E-06

Mean k for m selected sites (corrected for mutation rate)= 1.27999997E-03
Mean S for m selected sites (corrected for mutation rate)= 0.726400018
Var(Ss)= 0.726400018 Cov(S,k)= 1.27999997E-03
Uncorrected estimate of mean D for selected sites= -0.684334457

Theta-w for selected sites 9.54387011E-04 Delta-theta-w= 0.986588240

Statistics for mixture of m neutral and selected sites
Mean k for mixture= 0.135704011
Mean S for mixture= 2.63659239
d= -0.210706979 f(S)= 7.88071454E-02

1st and 2nd derivs wrt Sn= -0.452881932 0.131972939
1st and 2nd derivs wrt Ss= -0.342054486 4.87186834E-02
Mixed 2nd derivative= 0.130462095
1st approx. correction term for mean D= 1.24889631E-02
2nd approx. term for mean D= -3.75329256E-02
Estimate of theoretical D= -0.750578523
Corrected estimate of mean D= -0.775622487

Theta-w= 3.46410996E-03 Delta-theta-w= 0.608257174
Ratio of mixture to neutral D= 0.833976090

Uncorrected mean frequency of deleterious alleles= 1.28000001E-05

Mean k for m selected sites (corrected for mutation rate)= 2.55999994E-03
Mean S for m selected sites (corrected for mutation rate)= 1.45280004
Var(Ss)= 1.45280004 Cov(S,k)= 2.55999994E-03
Uncorrected estimate of mean D for selected sites= -0.941850662

Theta-w for selected sites 1.90877402E-03 Delta-theta-w= 0.986588240

Statistics for mixture of m neutral and selected sites
Mean k for mixture= 0.136408001
Mean S for mixture= 3.03611255
d= -0.262494296 f(S)= 9.31793600E-02

1st and 2nd derivs wrt Sn= -0.352252275 8.19260329E-02
1st and 2nd derivs wrt Ss= -0.294197023 4.05127332E-02
Mixed 2nd derivative= 9.49015394E-02
1st approx. correction term for mean D= 2.64072306E-02
2nd approx. term for mean D= -3.38676609E-02
Estimate of theoretical D= -0.859923899
Corrected estimate of mean D= -0.867384315

Theta-w= 3.98902316E-03 Delta-theta-w= 0.658041596
Ratio of mixture to neutral D= 0.955470979

Uncorrected mean frequency of deleterious alleles= 2.56000003E-05

Mean k for m selected sites (corrected for mutation rate)= 5.11999987E-03
Mean S for m selected sites (corrected for mutation rate)= 2.90560007
Var(Ss)= 2.90560007 Cov(S,k)= 5.11999987E-03
Uncorrected estimate of mean D for selected sites= -1.26665974

Theta-w for selected sites 3.81754804E-03 Delta-theta-w= 0.986588240

Statistics for mixture of m neutral and selected sites
Mean k for mixture= 0.137816012
Mean S for mixture= 3.83515263
d= -0.366068840 f(S)= 0.123842865

1st and 2nd derivs wrt Sn= -0.229893610 3.51064354E-02
1st and 2nd derivs wrt Ss= -0.228743494 2.99516525E-02
Mixed 2nd derivative= 5.55178188E-02
1st approx. correction term for mean D= 4.47316281E-02
2nd approx. term for mean D= -3.15519013E-02
Estimate of theoretical D= -1.04022491
Corrected estimate of mean D= -1.02704525

Theta-w= 5.03884908E-03 Delta-theta-w= 0.726493061
Ratio of mixture to neutral D= 1.15580547

Uncorrected mean frequency of deleterious alleles= 5.12000006E-05

Mean k for m selected sites (corrected for mutation rate)= 1.02399997E-02
Mean S for m selected sites (corrected for mutation rate)= 5.81120014
Var(Ss)= 5.81120014 Cov(S,k)= 1.02399997E-02
Uncorrected estimate of mean D for selected sites= -1.64118016

Theta-w for selected sites 7.63509609E-03 Delta-theta-w= 0.986588240

Statistics for mixture of m neutral and selected sites
Mean k for mixture= 0.140632004
Mean S for mixture= 5.43323231
d= -0.573217988 f(S)= 0.192846179

1st and 2nd derivs wrt Sn= -0.118308745 7.79214129E-03
1st and 2nd derivs wrt Ss= -0.155530438 1.88541748E-02
Mixed 2nd derivative= 2.42414735E-02
1st approx. correction term for mean D= 6.30710125E-02
2nd approx. term for mean D= -3.68322767E-02
Estimate of theoretical D= -1.30531192
Corrected estimate of mean D= -1.27907324

Theta-w= 7.13849999E-03 Delta-theta-w= 0.802995026
Ratio of mixture to neutral D= 1.45034647

Uncorrected mean frequency of deleterious alleles= 1.02400001E-04

Mean k for m selected sites (corrected for mutation rate)= 2.04799995E-02
Mean S for m selected sites (corrected for mutation rate)= 11.6224003
Var(Ss)= 11.6224003 Cov(S,k)= 2.04799995E-02
Uncorrected estimate of mean D for selected sites= -2.01921654

Theta-w for selected sites 1.52701922E-02 Delta-theta-w= 0.986588240

Statistics for mixture of m neutral and selected sites
Mean k for mixture= 0.146264002
Mean S for mixture= 8.62939262
d= -0.987516224 f(S)= 0.361558199

1st and 2nd derivs wrt Sn= -4.60856855E-02 -3.16464109E-04
1st and 2nd derivs wrt Ss= -9.00445208E-02 9.55652818E-03
Mixed 2nd derivative= 7.48572219E-03
1st approx. correction term for mean D= 7.40788728E-02
2nd approx. term for mean D= -5.44617064E-02
Estimate of theoretical D= -1.64231002
Corrected estimate of mean D= -1.62269282

Theta-w= 1.13378027E-02 Delta-theta-w= 0.870994389
Ratio of mixture to neutral D= 1.82478881

Observed Tajima D for neutral sites = -0.899999976
Neutral diversity= 6.00000005E-03

Equilibrium neutral values of k and S for m neutral sites
kn= 0.600000024 Sn= 4.56670141

Iteration= 1

f(S)= 0.154160082 derivative= 4.29084152E-02
g(S)= 2.54691267 derivative= -0.354449689
2nd derivative of g(S)= 0.114878483
3rd derivative of g(S)= -6.15079738E-02

Equilibrium Var(S)= 5.15856075 Cov(S,k)= 0.780317187
1st approx. correction term for mean D= -3.63506414E-02
2nd approx. correction term for mean D= 0.00000000
Coefficients of quadratic for S
a= 1.57675929E-02 b= -0.176016748 c= 0.360000014 discrim= 9.09756050E-02

Estimate of neutral mean S= 8.46649075
Theta-w= 1.11237727E-02 Delta-theta-w= 0.460614622
Check on D= -0.900000215

f(S)= 0.347376406 derivative= 5.82223274E-02
g(S)= 1.69667959 derivative= -0.142186731
2nd derivative of g(S)= 2.59596519E-02
3nd derivative of g(S)= -7.59664038E-03

Equilibrium Var(S)= 5.15856075 Cov(S,k)= 0.780317187
1st approx. correction term for mean D= -1.45819820E-02
2nd approx. correction term for mean D= -3.36152837E-02
Coefficients of quadratic for S
a= 1.58083159E-02 b= -0.175516680 c= 0.360000014 discrim= 8.96779299E-02

Estimate of neutral mean S= 8.38781929
Theta-w= 1.10204099E-02 Delta-theta-w= 0.455555558
Check on D= -0.900000036

Singletons only are assumed for selected sites

Uncorrected mean frequency of deleterious alleles= 1.00000001E-07

Mean k for m selected sites (corrected for mutation rate)= 1.99999995E-05
Mean S for m selected sites (corrected for mutation rate)= 1.13500003E-02
Var(Ss)= 1.13500003E-02 Cov(S,k)= 1.99999995E-05
Uncorrected estimate of mean D for selected sites= -8.79949182E-02

Theta-w for selected sites 1.49122970E-05 Delta-theta-w= 0.986588240

Statistics for mixture of m neutral and selected sites
Mean k for mixture= 0.270011008
Mean S for mixture= 3.78076100
d= -0.226727575 f(S)= 0.121674396

1st and 2nd derivs wrt Sn= -0.308654904 7.84474984E-02
1st and 2nd derivs wrt Ss= -0.159729317 7.31248409E-04
Mixed 2nd derivative= 5.15915640E-02
1st approx. correction term for mean D= -1.41151194E-02
2nd approx. term for mean D= -4.58765663E-02
Estimate of theoretical D= -0.649987102
Corrected estimate of mean D= -0.709978759

Theta-w= 4.96738590E-03 Delta-theta-w= 0.456432402
Ratio of mixture to neutral D= 0.722207844

Uncorrected mean frequency of deleterious alleles= 2.00000002E-07

Mean k for m selected sites (corrected for mutation rate)= 3.99999990E-05
Mean S for m selected sites (corrected for mutation rate)= 2.27000006E-02
Var(Ss)= 2.27000006E-02 Cov(S,k)= 3.99999990E-05
Uncorrected estimate of mean D for selected sites= -0.124386184

Theta-w for selected sites 2.98245941E-05 Delta-theta-w= 0.986588240

Statistics for mixture of m neutral and selected sites
Mean k for mixture= 0.270022005
Mean S for mixture= 3.78700352
d= -0.227536768 f(S)= 0.121922672

1st and 2nd derivs wrt Sn= -0.307712615 7.80056044E-02
1st and 2nd derivs wrt Ss= -0.159535512 7.72930682E-04
Mixed 2nd derivative= 5.14240302E-02
1st approx. correction term for mean D= -1.39426971E-02
2nd approx. term for mean D= -4.57820147E-02
Estimate of theoretical D= -0.651642442
Corrected estimate of mean D= -0.711367190

Theta-w= 4.97558760E-03 Delta-theta-w= 0.457306325
Ratio of mixture to neutral D= 0.724047124

Uncorrected mean frequency of deleterious alleles= 4.00000005E-07

Mean k for m selected sites (corrected for mutation rate)= 7.99999980E-05
Mean S for m selected sites (corrected for mutation rate)= 4.54000011E-02
Var(Ss)= 4.54000011E-02 Cov(S,k)= 7.99999980E-05
Uncorrected estimate of mean D for selected sites= -0.175746575

Theta-w for selected sites 5.96491882E-05 Delta-theta-w= 0.986588240

Statistics for mixture of m neutral and selected sites
Mean k for mixture= 0.270043999
Mean S for mixture= 3.79948854
d= -0.229155123 f(S)= 0.122419685

1st and 2nd derivs wrt Sn= -0.305840582 7.71308094E-02
1st and 2nd derivs wrt Ss= -0.159149036 8.55436549E-04
Mixed 2nd derivative= 5.10911904E-02
1st approx. correction term for mean D= -1.35993697E-02
2nd approx. term for mean D= -4.55930233E-02
Estimate of theoretical D= -0.654943645
Corrected estimate of mean D= -0.714136064

Theta-w= 4.99199145E-03 Delta-theta-w= 0.459045529
Ratio of mixture to neutral D= 0.727715135

Uncorrected mean frequency of deleterious alleles= 8.00000009E-07

Mean k for m selected sites (corrected for mutation rate)= 1.59999996E-04
Mean S for m selected sites (corrected for mutation rate)= 9.08000022E-02
Var(Ss)= 9.08000022E-02 Cov(S,k)= 1.59999996E-04
Uncorrected estimate of mean D for selected sites= -0.248086691

Theta-w for selected sites 1.19298376E-04 Delta-theta-w= 0.986588240

Statistics for mixture of m neutral and selected sites
Mean k for mixture= 0.270088017
Mean S for mixture= 3.82445860
d= -0.232391834 f(S)= 0.123415589

1st and 2nd derivs wrt Sn= -0.302146077 7.54163638E-02
1st and 2nd derivs wrt Ss= -0.158380598 1.01702288E-03
Mixed 2nd derivative= 5.04342131E-02
1st approx. correction term for mean D= -1.29187247E-02
2nd approx. term for mean D= -4.52155806E-02
Estimate of theoretical D= -0.661509156
Corrected estimate of mean D= -0.719643474

Theta-w= 5.02479821E-03 Delta-theta-w= 0.462489843
Ratio of mixture to neutral D= 0.735010147

Uncorrected mean frequency of deleterious alleles= 1.60000002E-06

Mean k for m selected sites (corrected for mutation rate)= 3.19999992E-04
Mean S for m selected sites (corrected for mutation rate)= 0.181600004
Var(Ss)= 0.181600004 Cov(S,k)= 3.19999992E-04
Uncorrected estimate of mean D for selected sites= -0.349566966

Theta-w for selected sites 2.38596753E-04 Delta-theta-w= 0.986588240

Statistics for mixture of m neutral and selected sites
Mean k for mixture= 0.270176023
Mean S for mixture= 3.87439847
d= -0.238865227 f(S)= 0.125414863

1st and 2nd derivs wrt Sn= -0.294950068 7.21229613E-02
1st and 2nd derivs wrt Ss= -0.156861708 1.32699311E-03
Mixed 2nd derivative= 4.91542630E-02
1st approx. correction term for mean D= -1.15810316E-02
2nd approx. term for mean D= -4.44637649E-02
Estimate of theoretical D= -0.674494565
Corrected estimate of mean D= -0.730539322

Theta-w= 5.09041222E-03 Delta-theta-w= 0.469245315
Ratio of mixture to neutral D= 0.749438405

Uncorrected mean frequency of deleterious alleles= 3.20000004E-06

Mean k for m selected sites (corrected for mutation rate)= 6.39999984E-04
Mean S for m selected sites (corrected for mutation rate)= 0.363200009
Var(Ss)= 0.363200009 Cov(S,k)= 6.39999984E-04
Uncorrected estimate of mean D for selected sites= -0.490799010

Theta-w for selected sites 4.77193506E-04 Delta-theta-w= 0.986588240

Statistics for mixture of m neutral and selected sites
Mean k for mixture= 0.270352006
Mean S for mixture= 3.97427845
d= -0.251812041 f(S)= 0.129443422

1st and 2nd derivs wrt Sn= -0.281288534 6.60391152E-02
1st and 2nd derivs wrt Ss= -0.153893754 1.89740025E-03
Mixed 2nd derivative= 4.67233807E-02
1st approx. correction term for mean D= -8.99654441E-03
2nd approx. term for mean D= -4.29787673E-02
Estimate of theoretical D= -0.699900806
Corrected estimate of mean D= -0.751876116

Theta-w= 5.22164069E-03 Delta-theta-w= 0.482246995
Ratio of mixture to neutral D= 0.777667522

Uncorrected mean frequency of deleterious alleles= 6.40000007E-06

Mean k for m selected sites (corrected for mutation rate)= 1.27999997E-03
Mean S for m selected sites (corrected for mutation rate)= 0.726400018
Var(Ss)= 0.726400018 Cov(S,k)= 1.27999997E-03
Uncorrected estimate of mean D for selected sites= -0.684334457

Theta-w for selected sites 9.54387011E-04 Delta-theta-w= 0.986588240

Statistics for mixture of m neutral and selected sites
Mean k for mixture= 0.270704001
Mean S for mixture= 4.17403841
d= -0.277705699 f(S)= 0.137620494

1st and 2nd derivs wrt Sn= -0.256594479 5.56127355E-02
1st and 2nd derivs wrt Ss= -0.148224175 2.86368094E-03
Mixed 2nd derivative= 4.23281379E-02
1st approx. correction term for mean D= -4.16566106E-03
2nd approx. term for mean D= -4.01231758E-02
Estimate of theoretical D= -0.748588622
Corrected estimate of mean D= -0.792877436

Theta-w= 5.48409671E-03 Delta-theta-w= 0.506383598
Ratio of mixture to neutral D= 0.831765115

Uncorrected mean frequency of deleterious alleles= 1.28000001E-05

Mean k for m selected sites (corrected for mutation rate)= 2.55999994E-03
Mean S for m selected sites (corrected for mutation rate)= 1.45280004
Var(Ss)= 1.45280004 Cov(S,k)= 2.55999994E-03
Uncorrected estimate of mean D for selected sites= -0.941850662

Theta-w for selected sites 1.90877402E-03 Delta-theta-w= 0.986588240

Statistics for mixture of m neutral and selected sites
Mean k for mixture= 0.271408021
Mean S for mixture= 4.57355833
d= -0.329492927 f(S)= 0.154454395

1st and 2nd derivs wrt Sn= -0.215810016 4.00564931E-02
1st and 2nd derivs wrt Ss= -0.137853399 4.24926914E-03
Mixed 2nd derivative= 3.50760296E-02
1st approx. correction term for mean D= 4.31851391E-03
2nd approx. term for mean D= -3.50592323E-02

Estimate of theoretical D= -0.838389754
Corrected estimate of mean D= -0.869130433

Theta-w= 6.00900967E-03 Delta-theta-w= 0.548331499
Ratio of mixture to neutral D= 0.931544125

Uncorrected mean frequency of deleterious alleles= 2.56000003E-05

Mean k for m selected sites (corrected for mutation rate)= 5.11999987E-03
Mean S for m selected sites (corrected for mutation rate)= 2.90560007
Var(Ss)= 2.90560007 Cov(S,k)= 5.11999987E-03
Uncorrected estimate of mean D for selected sites= -1.26665974

Theta-w for selected sites 3.81754804E-03 Delta-theta-w= 0.986588240

Statistics for mixture of m neutral and selected sites
Mean k for mixture= 0.272816002
Mean S for mixture= 5.37259865
d= -0.433067560 f(S)= 0.190041289

1st and 2nd derivs wrt Sn= -0.158124819 2.18153801E-02
1st and 2nd derivs wrt Ss= -0.120332599 5.63382357E-03
Mixed 2nd derivative= 2.48844810E-02
1st approx. correction term for mean D= 1.76293626E-02
2nd approx. term for mean D= -2.79124156E-02
Estimate of theoretical D= -0.993417203
Corrected estimate of mean D= -1.00370026

Theta-w= 7.05883559E-03 Delta-theta-w= 0.613511324
Ratio of mixture to neutral D= 1.10379684

Uncorrected mean frequency of deleterious alleles= 5.12000006E-05

Mean k for m selected sites (corrected for mutation rate)= 1.02399997E-02
Mean S for m selected sites (corrected for mutation rate)= 5.81120014
Var(Ss)= 5.81120014 Cov(S,k)= 1.02399997E-02
Uncorrected estimate of mean D for selected sites= -1.64118016

Theta-w for selected sites 7.63509609E-03 Delta-theta-w= 0.986588240

Statistics for mixture of m neutral and selected sites
Mean k for mixture= 0.275632024
Mean S for mixture= 6.97067833
d= -0.640216649 f(S)= 0.268891364

1st and 2nd derivs wrt Sn= -9.39521492E-02 7.26451539E-03
1st and 2nd derivs wrt Ss= -9.44640636E-02 5.97742014E-03
Mixed 2nd derivative= 1.38064828E-02
1st approx. correction term for mean D= 3.48004252E-02
2nd approx. term for mean D= -2.31151599E-02
Estimate of theoretical D= -1.23463488
Corrected estimate of mean D= -1.22294962

Theta-w= 9.15848650E-03 Delta-theta-w= 0.699041963
Ratio of mixture to neutral D= 1.37181652

Uncorrected mean frequency of deleterious alleles=   1.02400001E-04

Mean k for m selected sites (corrected for mutation rate)=   2.04799995E-02
Mean S for m selected sites (corrected for mutation rate)=   11.6224003
Var(Ss)=   11.6224003   Cov(S,k)=   2.04799995E-02
Uncorrected estimate of mean D for selected sites=   -2.01921654

Theta-w for selected sites   1.52701922E-02 Delta-theta-w=   0.986588240

Statistics for mixture of m neutral and selected sites
Mean k for mixture=   0.281264007
Mean S for mixture=   10.1668386
d=   -1.05451488   f(S)=   0.457296938

1st and 2nd derivs wrt Sn=   -4.23618145E-02   7.24618090E-04
1st and 2nd derivs wrt Ss=   -6.33034185E-02   4.56576189E-03
Mixed 2nd derivative=   5.44029148E-03
1st approx. correction term for mean D=   5.04980050E-02
2nd approx. term for mean D=   -2.99498569E-02
Estimate of theoretical D=   -1.55938578
Corrected estimate of mean D=   -1.53883767

Theta-w=   1.33577902E-02 Delta-theta-w=   0.789438188
Ratio of mixture to neutral D=   1.73265076

Observed Tajima D for neutral sites = -0.449999988

Neutral diversity=   3.00000003E-03

Equilibrium neutral values of k and S for m neutral sites
kn=   0.300000012   Sn=   2.28335071

Iteration=   1

f(S)=   6.66325763E-02 derivative=   3.37574258E-02
g(S)=   3.87397385   derivative= -0.981317163
2nd derivative of g(S)=   0.629230559
3nd derivative of g(S)= -0.678907394

Iteration=   5

f(S)=   0.100574650   derivative=   3.75715718E-02
g(S)=   3.15323067   derivative= -0.588974595
2nd derivative of g(S)=   0.267208934
3nd derivative of g(S)= -0.202613503

Equilibrium Var(S)=   2.43131542   Cov(S,k)=   0.345079303
1st approx. correction term for mean D=   -1.51006132E-02
2nd approx. correction term for mean D=   -4.06174995E-02
Coefficients of quadratic for S
a=   1.69507321E-02 b=   -8.26567933E-02 c=   9.00000036E-02 discrim=   2.70163231E-02

Estimate of neutral mean S=   3.23505545
Theta-w=   4.25040582E-03 Delta-theta-w=   0.294184983

Check on D= -0.449999839

Uncorrected mean frequency of deleterious alleles= 1.00000001E-07

Mean k for m selected sites (corrected for mutation rate)= 1.99999995E-05
Mean S for m selected sites (corrected for mutation rate)= 1.13500003E-02
Var(Ss)= 1.13500003E-02 Cov(S,k)= 1.99999995E-05
Uncorrected estimate of mean D for selected sites= -8.79949182E-02

Theta-w for selected sites 1.49122970E-05 Delta-theta-w= 0.986588240

Statistics for mixture of m neutral and selected sites
Mean k for mixture= 0.135011002
Mean S for mixture= 1.46201742
d= -5.70774376E-02 f(S)= 4.02582549E-02

1st and 2nd derivs wrt Sn= -1.04654872 0.547642827
1st and 2nd derivs wrt Ss= -0.839268684 0.287544012
Mixed 2nd derivative= 0.528699934
1st approx. correction term for mean D= -1.13954060E-02
2nd approx. term for mean D= -3.80922258E-02
Estimate of theoretical D= -0.284470350
Corrected estimate of mean D= -0.333957970

Theta-w= 1.92088436E-03 Delta-theta-w= 0.297141433
Ratio of mixture to neutral D= 0.632156551

Uncorrected mean frequency of deleterious alleles= 2.00000002E-07

Mean k for m selected sites (corrected for mutation rate)= 3.99999990E-05
Mean S for m selected sites (corrected for mutation rate)= 2.27000006E-02
Var(Ss)= 2.27000006E-02 Cov(S,k)= 3.99999990E-05
Uncorrected estimate of mean D for selected sites= -0.124386184

Theta-w for selected sites 2.98245941E-05 Delta-theta-w= 0.986588240

Statistics for mixture of m neutral and selected sites
Mean k for mixture= 0.135021999
Mean S for mixture= 1.46825993
d= -5.78866154E-02 f(S)= 4.04485166E-02

1st and 2nd derivs wrt Sn= -1.03917336 0.540704072
1st and 2nd derivs wrt Ss= -0.834891737 0.285085052
Mixed 2nd derivative= 0.523468673
1st approx. correction term for mean D= -1.06382798E-02
2nd approx. term for mean D= -3.82368192E-02
Estimate of theoretical D= -0.287823886
Corrected estimate of mean D= -0.336698979

Theta-w= 1.92908617E-03 Delta-theta-w= 0.300072730
Ratio of mixture to neutral D= 0.639608860

Uncorrected mean frequency of deleterious alleles= 4.00000005E-07

Mean k for m selected sites (corrected for mutation rate)= 7.99999980E-05
Mean S for m selected sites (corrected for mutation rate)= 4.54000011E-02

Var(Ss)= 4.54000011E-02 Cov(S,k)= 7.99999980E-05
Uncorrected estimate of mean D for selected sites= -0.175746575

Theta-w for selected sites 5.96491882E-05 Delta-theta-w= 0.986588240

Statistics for mixture of m neutral and selected sites
Mean k for mixture= 0.135044008
Mean S for mixture= 1.48074496
d= -5.95049560E-02 f(S)= 4.08295095E-02

1st and 2nd derivs wrt Sn= -1.02466202 0.527158678
1st and 2nd derivs wrt Ss= -0.826265574 0.280266643
Mixed 2nd derivative= 0.513225973
1st approx. correction term for mean D= -9.14762728E-03
2nd approx. term for mean D= -3.85119990E-02
Estimate of theoretical D= -0.294486970
Corrected estimate of mean D= -0.342146575

Theta-w= 1.94548978E-03 Delta-theta-w= 0.305861115
Ratio of mixture to neutral D= 0.654415727

Uncorrected mean frequency of deleterious alleles= 8.00000009E-07

Mean k for m selected sites (corrected for mutation rate)= 1.59999996E-04
Mean S for m selected sites (corrected for mutation rate)= 9.08000022E-02
Var(Ss)= 9.08000022E-02 Cov(S,k)= 1.59999996E-04
Uncorrected estimate of mean D for selected sites= -0.248086691

Theta-w for selected sites 1.19298376E-04 Delta-theta-w= 0.986588240

Statistics for mixture of m neutral and selected sites
Mean k for mixture= 0.135088012
Mean S for mixture= 1.50571489
d= -6.27416521E-02 f(S)= 4.15933579E-02

1st and 2nd derivs wrt Sn= -0.996565700 0.501336038
1st and 2nd derivs wrt Ss= -0.809507847 0.271011412
Mixed 2nd derivative= 0.493583053
1st approx. correction term for mean D= -6.25756802E-03
2nd approx. term for mean D= -3.90100554E-02
Estimate of theoretical D= -0.307640821
Corrected estimate of mean D= -0.352908432

Theta-w= 1.97829679E-03 Delta-theta-w= 0.317149878
Ratio of mixture to neutral D= 0.683646500

Uncorrected mean frequency of deleterious alleles= 1.60000002E-06

Mean k for m selected sites (corrected for mutation rate)= 3.19999992E-04
Mean S for m selected sites (corrected for mutation rate)= 0.181600004
Var(Ss)= 0.181600004 Cov(S,k)= 3.19999992E-04
Uncorrected estimate of mean D for selected sites= -0.349566966

Theta-w for selected sites 2.38596753E-04 Delta-theta-w= 0.986588240

Statistics for mixture of m neutral and selected sites

Mean k for mixture= 0.135176003
Mean S for mixture= 1.55565488
d= -6.92150742E-02 f(S)= 4.31285612E-02

1st and 2nd derivs wrt Sn= -0.943831384 0.454322457
1st and 2nd derivs wrt Ss= -0.777844846 0.253905445
Mixed 2nd derivative= 0.457394779
1st approx. correction term for mean D= -8.18805769E-04
2nd approx. term for mean D= -3.98232527E-02
Estimate of theoretical D= -0.333286852
Corrected estimate of mean D= -0.373928934

Theta-w= 2.04391079E-03 Delta-theta-w= 0.338640392
Ratio of mixture to neutral D= 0.740637720

Uncorrected mean frequency of deleterious alleles= 3.20000004E-06

Mean k for m selected sites (corrected for mutation rate)= 6.39999984E-04
Mean S for m selected sites (corrected for mutation rate)= 0.363200009
Var(Ss)= 0.363200009 Cov(S,k)= 6.39999984E-04
Uncorrected estimate of mean D for selected sites= -0.490799010

Theta-w for selected sites 4.77193506E-04 Delta-theta-w= 0.986588240

Statistics for mixture of m neutral and selected sites
Mean k for mixture= 0.135352001
Mean S for mixture= 1.65553486
d= -8.21618885E-02 f(S)= 4.62289527E-02

1st and 2nd derivs wrt Sn= -0.850493133 0.375852525
1st and 2nd derivs wrt Ss= -0.721057475 0.224484235
Mixed 2nd derivative= 0.395566016
1st approx. correction term for mean D= 8.85826722E-03
2nd approx. term for mean D= -4.08898629E-02
Estimate of theoretical D= -0.382131964
Corrected estimate of mean D= -0.414163560

Theta-w= 2.17513903E-03 Delta-theta-w= 0.377731681
Ratio of mixture to neutral D= 0.849182427

Uncorrected mean frequency of deleterious alleles= 6.40000007E-06

Mean k for m selected sites (corrected for mutation rate)= 1.27999997E-03
Mean S for m selected sites (corrected for mutation rate)= 0.726400018
Var(Ss)= 0.726400018 Cov(S,k)= 1.27999997E-03
Uncorrected estimate of mean D for selected sites= -0.684334457

Theta-w for selected sites 9.54387011E-04 Delta-theta-w= 0.986588240

Statistics for mixture of m neutral and selected sites
Mean k for mixture= 0.135704011
Mean S for mixture= 1.85529494
d= -0.108055532 f(S)= 5.25496863E-02

1st and 2nd derivs wrt Sn= -0.701756895 0.263816953
1st and 2nd derivs wrt Ss= -0.628186345 0.179893568

Mixed 2nd derivative= 0.303166717
1st approx. correction term for mean D= 2.44355686E-02
2nd approx. term for mean D= -4.17146683E-02
Estimate of theoretical D= -0.471370012
Corrected estimate of mean D= -0.488649100

Theta-w= 2.43759551E-03 Delta-theta-w= 0.443287373
Ratio of mixture to neutral D= 1.04748929

Uncorrected mean frequency of deleterious alleles= 1.28000001E-05

Mean k for m selected sites (corrected for mutation rate)= 2.55999994E-03
Mean S for m selected sites (corrected for mutation rate)= 1.45280004
Var(Ss)= 1.45280004 Cov(S,k)= 2.55999994E-03
Uncorrected estimate of mean D for selected sites= -0.941850662

Theta-w for selected sites 1.90877402E-03 Delta-theta-w= 0.986588240

Statistics for mixture of m neutral and selected sites
Mean k for mixture= 0.136408001
Mean S for mixture= 2.25481486
d= -0.159842819 f(S)= 6.56709075E-02

1st and 2nd derivs wrt Sn= -0.502322197 0.140404999
1st and 2nd derivs wrt Ss= -0.497231156 0.124585718
Mixed 2nd derivative= 0.192020893
1st approx. correction term for mean D= 4.57049794E-02
2nd approx. term for mean D= -4.17483076E-02
Estimate of theoretical D= -0.623744369
Corrected estimate of mean D= -0.619787693

Theta-w= 2.96250824E-03 Delta-theta-w= 0.539552331
Ratio of mixture to neutral D= 1.38609910

Uncorrected mean frequency of deleterious alleles= 2.56000003E-05

Mean k for m selected sites (corrected for mutation rate)= 5.11999987E-03
Mean S for m selected sites (corrected for mutation rate)= 2.90560007
Var(Ss)= 2.90560007 Cov(S,k)= 5.11999987E-03
Uncorrected estimate of mean D for selected sites= -1.26665974

Theta-w for selected sites 3.81754804E-03 Delta-theta-w= 0.986588240

Statistics for mixture of m neutral and selected sites
Mean k for mixture= 0.137816012
Mean S for mixture= 3.05385494
d= -0.263417393 f(S)= 9.38324556E-02

1st and 2nd derivs wrt Sn= -0.294111699 4.81193475E-02
1st and 2nd derivs wrt Ss= -0.346837163 7.22034127E-02
Mixed 2nd derivative= 9.37423185E-02
1st approx. correction term for mean D= 6.84537143E-02
2nd approx. term for mean D= -4.30407599E-02
Estimate of theoretical D= -0.859939516
Corrected estimate of mean D= -0.834526539

Theta-w= 4.01233416E-03 Delta-theta-w= 0.656519055
Ratio of mixture to neutral D= 1.91097736

Uncorrected mean frequency of deleterious alleles= 5.12000006E-05

Mean k for m selected sites (corrected for mutation rate)= 1.02399997E-02
Mean S for m selected sites (corrected for mutation rate)= 5.81120014
Var(Ss)= 5.81120014 Cov(S,k)= 1.02399997E-02
Uncorrected estimate of mean D for selected sites= -1.64118016

Theta-w for selected sites 7.63509609E-03 Delta-theta-w= 0.986588240

Statistics for mixture of m neutral and selected sites
Mean k for mixture= 0.140632004
Mean S for mixture= 4.65193510
d= -0.470566541 f(S)= 0.157831877

1st and 2nd derivs wrt Sn= -0.134818599 7.28208572E-03
1st and 2nd derivs wrt Ss= -0.210058302 3.50127630E-02
Mixed 2nd derivative= 3.37526798E-02
1st approx. correction term for mean D= 8.54713321E-02
2nd approx. term for mean D= -5.20378910E-02
Estimate of theoretical D= -1.18446898
Corrected estimate of mean D= -1.15103543

Theta-w= 6.11198554E-03 Delta-theta-w= 0.769907832
Ratio of mixture to neutral D= 2.63215423

Uncorrected mean frequency of deleterious alleles= 1.02400001E-04

Mean k for m selected sites (corrected for mutation rate)= 2.04799995E-02
Mean S for m selected sites (corrected for mutation rate)= 11.6224003
Var(Ss)= 11.6224003 Cov(S,k)= 2.04799995E-02
Uncorrected estimate of mean D for selected sites= -2.01921654

Theta-w for selected sites 1.52701922E-02 Delta-theta-w= 0.986588240

Statistics for mixture of m neutral and selected sites
Mean k for mixture= 0.146264002
Mean S for mixture= 7.84809494
d= -0.884864748 f(S)= 0.316536009

1st and 2nd derivs wrt Sn= -4.74686846E-02 -1.26022939E-03
1st and 2nd derivs wrt Ss= -0.109923340 1.42058684E-02
Mixed 2nd derivative= 8.80704075E-03
1st approx. correction term for mean D= 9.05344337E-02
2nd approx. term for mean D= -7.16927424E-02
Estimate of theoretical D= -1.57277036
Corrected estimate of mean D= -1.55392861

Theta-w= 1.03112878E-02 Delta-theta-w= 0.858151555
Ratio of mixture to neutral D= 3.49504638

Observed Tajima D for neutral sites = -0.449999988
Neutral diversity= 6.00000005E-03

Equilibrium neutral values of k and S for m neutral sites
kn= 0.600000024 Sn= 4.56670141

Iteration= 1

f(S)= 0.154160082 derivative= 4.29084152E-02
g(S)= 2.54691267 derivative= -0.354449689
2nd derivative of g(S)= 0.114878483
3rd derivative of g(S)= -6.15079738E-02

Estimate of neutral mean S= 6.05098772
Theta-w= 7.95014296E-03 Delta-theta-w= 0.245296538
Check on D= -0.450000346

Iteration= 5

f(S)= 0.218852341 derivative= 4.85763997E-02
g(S)= 2.13758993 derivative= -0.237229422
2nd derivative of g(S)= 5.94108887E-02
3rd derivative of g(S)= -2.42786035E-02

Estimate of neutral mean S= 5.98097467
Theta-w= 7.85815623E-03 Delta-theta-w= 0.236461997
Check on D= -0.450000495

Uncorrected mean frequency of deleterious alleles= 1.00000001E-07

Mean k for m selected sites (corrected for mutation rate)= 1.99999995E-05
Mean S for m selected sites (corrected for mutation rate)= 1.13500003E-02
Var(Ss)= 1.13500003E-02 Cov(S,k)= 1.99999995E-05
Uncorrected estimate of mean D for selected sites= -8.79949182E-02

Theta-w for selected sites 1.49122970E-05 Delta-theta-w= 0.986588240

Statistics for mixture of m neutral and selected sites
Mean k for mixture= 0.270011008
Mean S for mixture= 2.69768095
d= -8.44261646E-02 f(S)= 8.09632987E-02

1st and 2nd derivs wrt Sn= -0.474433720 0.152997166
1st and 2nd derivs wrt Ss= -0.294273525 2.60804482E-02
Mixed 2nd derivative= 0.119176894
1st approx. correction term for mean D= -2.16569118E-02
2nd approx. term for mean D= -3.33289653E-02
Estimate of theoretical D= -0.296710521
Corrected estimate of mean D= -0.351696372

Theta-w= 3.54437181E-03 Delta-theta-w= 0.238197803
Ratio of mixture to neutral D= 0.659355998

Uncorrected mean frequency of deleterious alleles= 2.00000002E-07

Mean k for m selected sites (corrected for mutation rate)= 3.99999990E-05
Mean S for m selected sites (corrected for mutation rate)= 2.27000006E-02
Var(Ss)= 2.27000006E-02 Cov(S,k)= 3.99999990E-05

Uncorrected estimate of mean D for selected sites= -0.124386184

Theta-w for selected sites 2.98245941E-05 Delta-theta-w= 0.986588240

Statistics for mixture of m neutral and selected sites
Mean k for mixture= 0.270022005
Mean S for mixture= 2.70392346
d= -8.52353275E-02 f(S)= 8.11844692E-02

1st and 2nd derivs wrt Sn= -0.472496271 0.151851088
1st and 2nd derivs wrt Ss= -0.293612570 2.60445848E-02
Mixed 2nd derivative= 0.118585289
1st approx. correction term for mean D= -2.13304497E-02
2nd approx. term for mean D= -3.34090218E-02
Estimate of theoretical D= -0.299145967
Corrected estimate of mean D= -0.353885442

Theta-w= 3.55257350E-03 Delta-theta-w= 0.239925623
Ratio of mixture to neutral D= 0.664768100

Uncorrected mean frequency of deleterious alleles= 4.00000005E-07

Mean k for m selected sites (corrected for mutation rate)= 7.99999980E-05
Mean S for m selected sites (corrected for mutation rate)= 4.54000011E-02
Var(Ss)= 4.54000011E-02 Cov(S,k)= 7.99999980E-05
Uncorrected estimate of mean D for selected sites= -0.175746575

Theta-w for selected sites 5.96491882E-05 Delta-theta-w= 0.986588240

Statistics for mixture of m neutral and selected sites
Mean k for mixture= 0.270043999
Mean S for mixture= 2.71640849
d= -8.68537128E-02 f(S)= 8.16272944E-02

1st and 2nd derivs wrt Sn= -0.468656600 0.149590194
1st and 2nd derivs wrt Ss= -0.292299330 2.59729624E-02
Mixed 2nd derivative= 0.117414437
1st approx. correction term for mean D= -2.06823312E-02
2nd approx. term for mean D= -3.35624143E-02
Estimate of theoretical D= -0.303997964
Corrected estimate of mean D= -0.358242720

Theta-w= 3.56897712E-03 Delta-theta-w= 0.243357420
Ratio of mixture to neutral D= 0.675550282

Uncorrected mean frequency of deleterious alleles= 8.00000009E-07

Mean k for m selected sites (corrected for mutation rate)= 1.59999996E-04
Mean S for m selected sites (corrected for mutation rate)= 9.08000022E-02
Var(Ss)= 9.08000022E-02 Cov(S,k)= 1.59999996E-04
Uncorrected estimate of mean D for selected sites= -0.248086691

Theta-w for selected sites 1.19298376E-04 Delta-theta-w= 0.986588240

Statistics for mixture of m neutral and selected sites
Mean k for mixture= 0.270088017

Mean S for mixture= 2.74137855
d= -9.00903940E-02 f(S)= 8.25148001E-02

1st and 2nd derivs wrt Sn= -0.461115837 0.145191044
1st and 2nd derivs wrt Ss= -0.289707154 2.58302875E-02
Mixed 2nd derivative= 0.115121506
1st approx. correction term for mean D= -1.94050204E-02
2nd approx. term for mean D= -3.38434577E-02
Estimate of theoretical D= -0.313626379
Corrected estimate of mean D= -0.366874844

Theta-w= 3.60178412E-03 Delta-theta-w= 0.250127196
Ratio of mixture to neutral D= 0.696946740

Uncorrected mean frequency of deleterious alleles= 1.60000002E-06

Mean k for m selected sites (corrected for mutation rate)= 3.19999992E-04
Mean S for m selected sites (corrected for mutation rate)= 0.181600004
Var(Ss)= 0.181600004 Cov(S,k)= 3.19999992E-04
Uncorrected estimate of mean D for selected sites= -0.349566966

Theta-w for selected sites 2.38596753E-04 Delta-theta-w= 0.986588240

Statistics for mixture of m neutral and selected sites
Mean k for mixture= 0.270176023
Mean S for mixture= 2.79131842
d= -9.65637863E-02 f(S)= 8.42973143E-02

1st and 2nd derivs wrt Sn= -0.446567625 0.136857897
1st and 2nd derivs wrt Ss= -0.284655660 2.55471617E-02
Mixed 2nd derivative= 0.110722326
1st approx. correction term for mean D= -1.69236287E-02
2nd approx. term for mean D= -3.43105197E-02
Estimate of theoretical D= -0.332588643
Corrected estimate of mean D= -0.383822799

Theta-w= 3.66739812E-03 Delta-theta-w= 0.263303220
Ratio of mixture to neutral D= 0.739085078

Uncorrected mean frequency of deleterious alleles= 3.20000004E-06

Mean k for m selected sites (corrected for mutation rate)= 6.39999984E-04
Mean S for m selected sites (corrected for mutation rate)= 0.363200009
Var(Ss)= 0.363200009 Cov(S,k)= 6.39999984E-04
Uncorrected estimate of mean D for selected sites= -0.490799010

Theta-w for selected sites 4.77193506E-04 Delta-theta-w= 0.986588240

Statistics for mixture of m neutral and selected sites
Mean k for mixture= 0.270352006
Mean S for mixture= 2.89119840
d= -0.109510630 f(S)= 8.78923312E-02

1st and 2nd derivs wrt Sn= -0.419451118 0.121874213
1st and 2nd derivs wrt Ss= -0.275051773 2.49899141E-02
Mixed 2nd derivative= 0.102610677

1st approx. correction term for mean D= -1.22355018E-02
2nd approx. term for mean D= -3.49214002E-02
Estimate of theoretical D= -0.369386286
Corrected estimate of mean D= -0.416543186

Theta-w= 3.79862636E-03 Delta-theta-w= 0.288290024
Ratio of mixture to neutral D= 0.820857525

Uncorrected mean frequency of deleterious alleles= 6.40000007E-06

Mean k for m selected sites (corrected for mutation rate)= 1.27999997E-03
Mean S for m selected sites (corrected for mutation rate)= 0.726400018
Var(Ss)= 0.726400018 Cov(S,k)= 1.27999997E-03
Uncorrected estimate of mean D for selected sites= -0.684334457

Theta-w for selected sites 9.54387011E-04 Delta-theta-w= 0.986588240

Statistics for mixture of m neutral and selected sites
Mean k for mixture= 0.270704001
Mean S for mixture= 3.09095836
d= -0.135404259 f(S)= 9.52022970E-02

1st and 2nd derivs wrt Sn= -0.372080296 9.74521562E-02
1st and 2nd derivs wrt Ss= -0.257615834 2.39118524E-02
Mixed 2nd derivative= 8.87266770E-02
1st approx. correction term for mean D= -3.83021403E-03
2nd approx. term for mean D= -3.52106877E-02
Estimate of theoretical D= -0.438842446
Corrected estimate of mean D= -0.477883369

Theta-w= 4.06108284E-03 Delta-theta-w= 0.333419085
Ratio of mixture to neutral D= 0.975204349

Uncorrected mean frequency of deleterious alleles= 1.28000001E-05

Mean k for m selected sites (corrected for mutation rate)= 2.55999994E-03
Mean S for m selected sites (corrected for mutation rate)= 1.45280004
Var(Ss)= 1.45280004 Cov(S,k)= 2.55999994E-03
Uncorrected estimate of mean D for selected sites= -0.941850662

Theta-w for selected sites 1.90877402E-03 Delta-theta-w= 0.986588240

Statistics for mixture of m neutral and selected sites
Mean k for mixture= 0.271408021
Mean S for mixture= 3.49047852
d= -0.187191546 f(S)= 0.110302031

1st and 2nd derivs wrt Sn= -0.298354477 6.40755519E-02
1st and 2nd derivs wrt Ss= -0.228424460 2.19022147E-02
Mixed 2nd derivative= 6.79028779E-02
1st approx. correction term for mean D= 9.88996122E-03
2nd approx. term for mean D= -3.39150950E-02
Estimate of theoretical D= -0.563630462
Corrected estimate of mean D= -0.587655604

Theta-w= 4.58599580E-03 Delta-theta-w= 0.408180773

Ratio of mixture to neutral D= 1.25251079

Uncorrected mean frequency of deleterious alleles= 2.56000003E-05

Mean k for m selected sites (corrected for mutation rate)= 5.11999987E-03
Mean S for m selected sites (corrected for mutation rate)= 2.90560007
Var(Ss)= 2.90560007 Cov(S,k)= 5.11999987E-03
Uncorrected estimate of mean D for selected sites= -1.26665974

Theta-w for selected sites 3.81754804E-03 Delta-theta-w= 0.986588240

Statistics for mixture of m neutral and selected sites
Mean k for mixture= 0.272816002
Mean S for mixture= 4.28951836
d= -0.290766120 f(S)= 0.142420545

1st and 2nd derivs wrt Sn= -0.203352183 3.00400089E-02
1st and 2nd derivs wrt Ss= -0.185479641 1.84438936E-02
Mixed 2nd derivative= 4.27024439E-02
1st approx. correction term for mean D= 2.90372856E-02
2nd approx. term for mean D= -3.03201880E-02
Estimate of theoretical D= -0.770473063
Corrected estimate of mean D= -0.771755934

Theta-w= 5.63582126E-03 Delta-theta-w= 0.515925050
Ratio of mixture to neutral D= 1.71216047

Uncorrected mean frequency of deleterious alleles= 5.12000006E-05

Mean k for m selected sites (corrected for mutation rate)= 1.02399997E-02
Mean S for m selected sites (corrected for mutation rate)= 5.81120014
Var(Ss)= 5.81120014 Cov(S,k)= 1.02399997E-02
Uncorrected estimate of mean D for selected sites= -1.64118016

Theta-w for selected sites 7.63509609E-03 Delta-theta-w= 0.986588240

Statistics for mixture of m neutral and selected sites
Mean k for mixture= 0.275632024
Mean S for mixture= 5.88759851
d= -0.497915268 f(S)= 0.214333922

1st and 2nd derivs wrt Sn= -0.110146694 7.76293874E-03
1st and 2nd derivs wrt Ss= -0.132738099 1.33644743E-02
Mixed 2nd derivative= 2.03064401E-02
1st approx. correction term for mean D= 4.99099195E-02
2nd approx. term for mean D= -2.93046124E-02
Estimate of theoretical D= -1.07549894
Corrected estimate of mean D= -1.05489361

Theta-w= 7.73547264E-03 Delta-theta-w= 0.643677831
Ratio of mixture to neutral D= 2.38999510

Uncorrected mean frequency of deleterious alleles= 1.02400001E-04

Mean k for m selected sites (corrected for mutation rate)= 2.04799995E-02

Mean S for m selected sites (corrected for mutation rate)= 11.6224003
Var(Ss)= 11.6224003 Cov(S,k)= 2.04799995E-02
Uncorrected estimate of mean D for selected sites= -2.01921654

Theta-w for selected sites 1.52701922E-02 Delta-theta-w= 0.986588240

Statistics for mixture of m neutral and selected sites
Mean k for mixture= 0.281264007
Mean S for mixture= 9.08375835
d= -0.912213504 f(S)= 0.388866007

1st and 2nd derivs wrt Sn= -4.50721085E-02 8.18790868E-05
1st and 2nd derivs wrt Ss= -8.07280838E-02 7.64693366E-03
Mixed 2nd derivative= 6.80696825E-03
1st approx. correction term for mean D= 6.48109466E-02
2nd approx. term for mean D= -4.07294706E-02
Estimate of theoretical D= -1.46283889
Corrected estimate of mean D= -1.43875742

Theta-w= 1.19347759E-02 Delta-theta-w= 0.764332354
Ratio of mixture to neutral D= 3.25074959

Observed Tajima D for neutral sites = -0.224999994
Neutral diversity= 3.00000003E-03

Equilibrium neutral values of k and S for m neutral sites
kn= 0.300000012 Sn= 2.28335071
Iteration= 1

f(S)= 6.66325763E-02 derivative= 3.37574258E-02
g(S)= 3.87397385 derivative= -0.981317163
2nd derivative of g(S)= 0.629230559
3rd derivative of g(S)= -0.678907394

Equilibrium Var(S)= 2.43131542 Cov(S,k)= 0.345079303
1st approx. correction term for mean D= -2.51598135E-02
2nd approx. correction term for mean D= 0.00000000
Coefficients of quadratic for S
a= 1.71822198E-02 b= -7.98142105E-02 c= 9.00000036E-02 discrim= 1.35907764E-02

Estimate of neutral mean S= 2.71807098
Theta-w= 3.57116130E-03 Delta-theta-w= 0.159937084
Check on D= -0.225000590

Iteration= 5

f(S)= 7.99009353E-02 derivative= 3.52975205E-02
g(S)= 3.53772473 derivative= -0.781423390
2nd derivative of g(S)= 0.429086149
3nd derivative of g(S)= -0.395499408

Equilibrium Var(S)= 2.43131542 Cov(S,k)= 0.345079303
1st approx. correction term for mean D= -2.00347733E-02
2nd approx. correction term for mean D= -2.63364371E-02
Coefficients of quadratic for S
a= 1.71983074E-02 b= -7.96166733E-02 c= 9.00000036E-02 discrim= 1.21418163E-02

Estimate of neutral mean S=   2.66766047
Theta-w=   3.50492913E-03 Delta-theta-w=   0.144062459
Check on D= -0.225000128

Uncorrected mean frequency of deleterious alleles=   1.00000001E-07

Mean k for m selected sites (corrected for mutation rate)=   1.99999995E-05
Mean S for m selected sites (corrected for mutation rate)=   1.13500003E-02
Var(Ss)=   1.13500003E-02 Cov(S,k)=   1.99999995E-05
Uncorrected estimate of mean D for selected sites=   -8.79949182E-02

Theta-w for selected sites   1.49122970E-05 Delta-theta-w=   0.986588240

Statistics for mixture of m neutral and selected sites
Mean k for mixture=   0.135011002
Mean S for mixture=   1.20668972
d=   -2.35309750E-02 f(S)=   3.26101370E-02

1st and 2nd derivs wrt Sn=   -1.34864926   0.832235575
1st and 2nd derivs wrt Ss=   -1.15120995   0.530816436
Mixed 2nd derivative=   0.841106772
1st approx. correction term for mean D=   -1.46284653E-02
2nd approx. term for mean D=   -2.38774940E-02
Estimate of theoretical D= -0.130305752
Corrected estimate of mean D= -0.168811709

Theta-w=   1.58541987E-03 Delta-theta-w=   0.148421109
Ratio of mixture to neutral D=   0.579136372

Uncorrected mean frequency of deleterious alleles=   2.00000002E-07

Mean k for m selected sites (corrected for mutation rate)=   3.99999990E-05
Mean S for m selected sites (corrected for mutation rate)=   2.27000006E-02
Var(Ss)=   2.27000006E-02 Cov(S,k)=   3.99999990E-05
Uncorrected estimate of mean D for selected sites= -0.124386184

Theta-w for selected sites   2.98245941E-05 Delta-theta-w=   0.986588240

Statistics for mixture of m neutral and selected sites
Mean k for mixture=   0.135021999
Mean S for mixture=   1.21293223
d=   -2.43401527E-02 f(S)=   3.27940099E-02

1st and 2nd derivs wrt Sn=   -1.33732259   0.819767535
1st and 2nd derivs wrt Ss=   -1.14364779   0.524981618
Mixed 2nd derivative=   0.830896616
1st approx. correction term for mean D=   -1.35784913E-02
2nd approx. term for mean D=   -2.44013723E-02
Estimate of theoretical D= -0.134408295
Corrected estimate of mean D= -0.172388151

Theta-w=   1.59362168E-03 Delta-theta-w=   0.152734876
Ratio of mixture to neutral D=   0.597369850

Uncorrected mean frequency of deleterious alleles=   4.00000005E-07

Mean k for m selected sites (corrected for mutation rate)= 7.99999980E-05
Mean S for m selected sites (corrected for mutation rate)= 4.54000011E-02
Var(Ss)= 4.54000011E-02 Cov(S,k)= 7.99999980E-05
Uncorrected estimate of mean D for selected sites= -0.175746575

Theta-w for selected sites 5.96491882E-05 Delta-theta-w= 0.986588240

Statistics for mixture of m neutral and selected sites
Mean k for mixture= 0.135044008
Mean S for mixture= 1.22541726
d= -2.59585083E-02 f(S)= 3.31622250E-02

1st and 2nd derivs wrt Sn= -1.31511116 0.795543194
1st and 2nd derivs wrt Ss= -1.12879586 0.513603747
Mixed 2nd derivative= 0.811000228
1st approx. correction term for mean D= -1.15194740E-02
2nd approx. term for mean D= -2.54073311E-02
Estimate of theoretical D= -0.142546937
Corrected estimate of mean D= -0.179473743

Theta-w= 1.61002518E-03 Delta-theta-w= 0.161230505
Ratio of mixture to neutral D= 0.633541584

Uncorrected mean frequency of deleterious alleles= 8.00000009E-07

Mean k for m selected sites (corrected for mutation rate)= 1.59999996E-04
Mean S for m selected sites (corrected for mutation rate)= 9.08000022E-02
Var(Ss)= 9.08000022E-02 Cov(S,k)= 1.59999996E-04
Uncorrected estimate of mean D for selected sites= -0.248086691

Theta-w for selected sites 1.19298376E-04 Delta-theta-w= 0.986588240

Statistics for mixture of m neutral and selected sites
Mean k for mixture= 0.135088012
Mean S for mixture= 1.25038719
d= -2.91952044E-02 f(S)= 3.39005254E-02

1st and 2nd derivs wrt Sn= -1.27238429 0.749787569
1st and 2nd derivs wrt Ss= -1.10013855 0.491956770
Mixed 2nd derivative= 0.773197055
1st approx. correction term for mean D= -7.55844451E-03
2nd approx. term for mean D= -2.72630602E-02
Estimate of theoretical D= -0.158565357
Corrected estimate of mean D= -0.193386868

Theta-w= 1.64283218E-03 Delta-theta-w= 0.177712619
Ratio of mixture to neutral D= 0.704734504

Uncorrected mean frequency of deleterious alleles= 1.60000002E-06

Mean k for m selected sites (corrected for mutation rate)= 3.19999992E-04
Mean S for m selected sites (corrected for mutation rate)= 0.181600004
Var(Ss)= 0.181600004 Cov(S,k)= 3.19999992E-04
Uncorrected estimate of mean D for selected sites= -0.349566966

Theta-w for selected sites   2.38596753E-04 Delta-theta-w=   0.986588240

Statistics for mixture of m neutral and selected sites
Mean k for mixture=   0.135176003
Mean S for mixture=   1.30032718
d=   -3.56686115E-02 f(S)=   3.53846252E-02

1st and 2nd derivs wrt Sn=   -1.19318032   0.667942405
1st and 2nd derivs wrt Ss=   -1.04669118   0.452672839
Mixed 2nd derivative=   0.704779387
1st approx. correction term for mean D=   -2.14955769E-04
2nd approx. term for mean D=   -3.04286070E-02
Estimate of theoretical D= -0.189617708
Corrected estimate of mean D= -0.220261261

Theta-w=   1.70844630E-03 Delta-theta-w=   0.208778083
Ratio of mixture to neutral D=   0.842744887

Uncorrected mean frequency of deleterious alleles=   3.20000004E-06

Mean k for m selected sites (corrected for mutation rate)=   6.39999984E-04
Mean S for m selected sites (corrected for mutation rate)=   0.363200009
Var(Ss)=   0.363200009   Cov(S,k)=   6.39999984E-04
Uncorrected estimate of mean D for selected sites= -0.490799010

Theta-w for selected sites   4.77193506E-04 Delta-theta-w=   0.986588240

Statistics for mixture of m neutral and selected sites
Mean k for mixture=   0.135352001
Mean S for mixture=   1.40020716
d=   -4.86154407E-02 f(S)=   3.83828133E-02

1st and 2nd derivs wrt Sn=   -1.05614305   0.535679579
1st and 2nd derivs wrt Ss= -0.953094006   0.387338966
Mixed 2nd derivative=   0.591627240
1st approx. correction term for mean D=   1.24938590E-02
2nd approx. term for mean D=   -3.50781679E-02
Estimate of theoretical D= -0.248145163
Corrected estimate of mean D= -0.270729482

Theta-w=   1.83967443E-03 Delta-theta-w=   0.264261067
Ratio of mixture to neutral D=   1.10286677

Uncorrected mean frequency of deleterious alleles=   6.40000007E-06

Mean k for m selected sites (corrected for mutation rate)=   1.27999997E-03
Mean S for m selected sites (corrected for mutation rate)=   0.726400018
Var(Ss)=   0.726400018   Cov(S,k)=   1.27999997E-03
Uncorrected estimate of mean D for selected sites= -0.684334457

Theta-w for selected sites   9.54387011E-04 Delta-theta-w=   0.986588240

Statistics for mixture of m neutral and selected sites
Mean k for mixture=   0.135704011
Mean S for mixture=   1.59996724
d=   -7.45090842E-02 f(S)=   4.44991365E-02

1st and 2nd derivs wrt Sn= -0.846058905 0.356938303
1st and 2nd derivs wrt Ss= -0.806150913 0.293863654
Mixed 2nd derivative= 0.431632340
1st approx. correction term for mean D= 3.19869891E-02
2nd approx. term for mean D= -4.02830429E-02
Estimate of theoretical D= -0.353210360
Corrected estimate of mean D= -0.361506432

Theta-w= 2.10213102E-03 Delta-theta-w= 0.354445457
Ratio of mixture to neutral D= 1.56982291

Uncorrected mean frequency of deleterious alleles= 1.28000001E-05

Mean k for m selected sites (corrected for mutation rate)= 2.55999994E-03
Mean S for m selected sites (corrected for mutation rate)= 1.45280004
Var(Ss)= 1.45280004 Cov(S,k)= 2.55999994E-03
Uncorrected estimate of mean D for selected sites= -0.941850662

Theta-w for selected sites 1.90877402E-03 Delta-theta-w= 0.986588240

Statistics for mixture of m neutral and selected sites
Mean k for mixture= 0.136408001
Mean S for mixture= 1.99948716
d= -0.126296371 f(S)= 5.72115406E-02

1st and 2nd derivs wrt Sn= -0.580366731 0.175800234
1st and 2nd derivs wrt Ss= -0.611494243 0.187778488
Mixed 2nd derivative= 0.254658401
1st approx. correction term for mean D= 5.66392913E-02
2nd approx. term for mean D= -4.42182794E-02
Estimate of theoretical D= -0.528018415
Corrected estimate of mean D= -0.515597403

Theta-w= 2.62704375E-03 Delta-theta-w= 0.480754733
Ratio of mixture to neutral D= 2.34674716

Uncorrected mean frequency of deleterious alleles= 2.56000003E-05

Mean k for m selected sites (corrected for mutation rate)= 5.11999987E-03
Mean S for m selected sites (corrected for mutation rate)= 2.90560007
Var(Ss)= 2.90560007 Cov(S,k)= 5.11999987E-03
Uncorrected estimate of mean D for selected sites= -1.26665974

Theta-w for selected sites 3.81754804E-03 Delta-theta-w= 0.986588240

Statistics for mixture of m neutral and selected sites
Mean k for mixture= 0.137816012
Mean S for mixture= 2.79852724
d= -0.229870945 f(S)= 8.45554471E-02

1st and 2nd derivs wrt Sn= -0.323009700 5.43425307E-02
1st and 2nd derivs wrt Ss= -0.405455172 9.85849053E-02
Mixed 2nd derivative= 0.114248492
1st approx. correction term for mean D= 8.02629814E-02
2nd approx. term for mean D= -4.81087863E-02

Estimate of theoretical D= -0.790520787
Corrected estimate of mean D= -0.758366585

Theta-w= 3.67686944E-03 Delta-theta-w= 0.625181079
Ratio of mixture to neutral D= 3.51342368

Uncorrected mean frequency of deleterious alleles= 5.12000006E-05

Mean k for m selected sites (corrected for mutation rate)= 1.02399997E-02
Mean S for m selected sites (corrected for mutation rate)= 5.81120014
Var(Ss)= 5.81120014 Cov(S,k)= 1.02399997E-02
Uncorrected estimate of mean D for selected sites= -1.64118016

Theta-w for selected sites 7.63509609E-03 Delta-theta-w= 0.986588240

Statistics for mixture of m neutral and selected sites
Mean k for mixture= 0.140632004
Mean S for mixture= 4.39660740
d= -0.437020093 f(S)= 0.146919578

1st and 2nd derivs wrt Sn= -0.141029015 6.85814768E-03
1st and 2nd derivs wrt Ss= -0.233890429 4.33343053E-02
Mixed 2nd derivative= 3.79299261E-02
1st approx. correction term for mean D= 9.52732861E-02
2nd approx. term for mean D= -5.86696491E-02
Estimate of theoretical D= -1.14014888
Corrected estimate of mean D= -1.10354531

Theta-w= 5.77652128E-03 Delta-theta-w= 0.756545484
Ratio of mixture to neutral D= 5.06732559

Uncorrected mean frequency of deleterious alleles= 1.02400001E-04

Mean k for m selected sites (corrected for mutation rate)= 2.04799995E-02
Mean S for m selected sites (corrected for mutation rate)= 11.6224003
Var(Ss)= 11.6224003 Cov(S,k)= 2.04799995E-02
Uncorrected estimate of mean D for selected sites= -2.01921654

Theta-w for selected sites 1.52701922E-02 Delta-theta-w= 0.986588240

Statistics for mixture of m neutral and selected sites
Mean k for mixture= 0.146264002
Mean S for mixture= 7.59276724
d= -0.851318359 f(S)= 0.302353173

1st and 2nd derivs wrt Sn= -4.77701053E-02 -1.65947410E-03
1st and 2nd derivs wrt Ss= -0.117747813 1.62418187E-02
Mixed 2nd derivative= 9.27870814E-03
1st approx. correction term for mean D= 9.70142856E-02
2nd approx. term for mean D= -7.86338225E-02
Estimate of theoretical D= -1.54822731
Corrected estimate of mean D= -1.52984679

Theta-w= 9.97582357E-03 Delta-theta-w= 0.853381515
Ratio of mixture to neutral D= 6.88100624

Observed Tajima D for neutral sites = -0.224999994
Neutral diversity= 6.00000005E-03

Equilibrium neutral values of k and S for m neutral sites
kn= 0.600000024 Sn= 4.56670141

Iteration= 1

f(S)= 0.154160082 derivative= 4.29084152E-02
g(S)= 2.54691267 derivative= -0.354449689
2nd derivative of g(S)= 0.114878483
3rd derivative of g(S)= -6.15079738E-02

Equilibrium Var(S)= 5.15856075 Cov(S,k)= 0.780317187
1st approx. correction term for mean D= -3.63506414E-02
2nd approx. correction term for mean D= 0.00000000
Coefficients of quadratic for S
a= 1.71909314E-02 b= -0.158538759 c= 0.360000014 discrim= 1.94832217E-02

Estimate of neutral mean S= 5.17778778
Theta-w= 6.80288160E-03 Delta-theta-w= 0.118020773
Check on D= -0.225000918

Iteration= 5

f(S)= 0.179411277 derivative= 4.52054292E-02
g(S)= 2.36088657 derivative= -0.297430843
2nd derivative of g(S)= 8.60445797E-02
3nd derivative of g(S)= -4.09125648E-02

Equilibrium Var(S)= 5.15856075 Cov(S,k)= 0.780317187
1st approx. correction term for mean D= -3.05030663E-02
2nd approx. correction term for mean D= -1.67124048E-02
Coefficients of quadratic for S
a= 1.71989109E-02 b= -0.158440799 c= 0.360000014 discrim= 1.83590557E-02

Estimate of neutral mean S= 5.13985586
Theta-w= 6.75304467E-03 Delta-theta-w= 0.111511767
Check on D= -0.225000620

Uncorrected mean frequency of deleterious alleles= 1.00000001E-07

Mean k for m selected sites (corrected for mutation rate)= 1.99999995E-05
Mean S for m selected sites (corrected for mutation rate)= 1.13500003E-02
Var(Ss)= 1.13500003E-02 Cov(S,k)= 1.99999995E-05
Uncorrected estimate of mean D for selected sites= -8.79949182E-02

Theta-w for selected sites 1.49122970E-05 Delta-theta-w= 0.986588240

Statistics for mixture of m neutral and selected sites
Mean k for mixture= 0.270011008
Mean S for mixture= 2.31917763
d= -3.46961617E-02 f(S)= 6.78445697E-02

1st and 2nd derivs wrt Sn= -0.575573325 0.207841367
1st and 2nd derivs wrt Ss= -0.383628458 5.26335761E-02

Mixed 2nd derivative=   0.172540173
1st approx. correction term for mean D=   -2.62521822E-02
2nd approx. term for mean D=   -1.86103210E-02
Estimate of theoretical D= -0.133206025
Corrected estimate of mean D= -0.178068519

Theta-w=   3.04707186E-03 Delta-theta-w=   0.113867223
Ratio of mixture to neutral D=   0.592025161

Uncorrected mean frequency of deleterious alleles=   2.00000002E-07

Mean k for m selected sites (corrected for mutation rate)=   3.99999990E-05
Mean S for m selected sites (corrected for mutation rate)=   2.27000006E-02
Var(Ss)=   2.27000006E-02 Cov(S,k)=   3.99999990E-05
Uncorrected estimate of mean D for selected sites= -0.124386184

Theta-w for selected sites   2.98245941E-05 Delta-theta-w=   0.986588240

Statistics for mixture of m neutral and selected sites
Mean k for mixture=   0.270022005
Mean S for mixture=   2.32542014
d=   -3.55053544E-02 f(S)=   6.80562779E-02

1st and 2nd derivs wrt Sn= -0.572889626   0.206070781
1st and 2nd derivs wrt Ss= -0.382544339   5.24535924E-02
Mixed 2nd derivative=   0.171517372
1st approx. correction term for mean D=   -2.58196630E-02
2nd approx. term for mean D=   -1.88927427E-02
Estimate of theoretical D= -0.136100516
Corrected estimate of mean D= -0.180812925

Theta-w=   3.05527356E-03 Delta-theta-w=   0.116210043
Ratio of mixture to neutral D=   0.604889512

Uncorrected mean frequency of deleterious alleles=   4.00000005E-07

Mean k for m selected sites (corrected for mutation rate)=   7.99999980E-05
Mean S for m selected sites (corrected for mutation rate)=   4.54000011E-02
Var(Ss)=   4.54000011E-02 Cov(S,k)=   7.99999980E-05
Uncorrected estimate of mean D for selected sites= -0.175746575

Theta-w for selected sites   5.96491882E-05 Delta-theta-w=   0.986588240

Statistics for mixture of m neutral and selected sites
Mean k for mixture=   0.270043999
Mean S for mixture=   2.33790517
d=   -3.71237099E-02 f(S)=   6.84801564E-02

1st and 2nd derivs wrt Sn= -0.567578793   0.202585280
1st and 2nd derivs wrt Ss= -0.380394131   5.20972982E-02
Mixed 2nd derivative=   0.169497609
1st approx. correction term for mean D=   -2.49624364E-02
2nd approx. term for mean D=   -1.94419418E-02
Estimate of theoretical D= -0.141862959
Corrected estimate of mean D= -0.186267346

Theta-w= 3.07167717E-03 Delta-theta-w= 0.120858133
Ratio of mixture to neutral D= 0.630500317

Uncorrected mean frequency of deleterious alleles= 8.00000009E-07

Mean k for m selected sites (corrected for mutation rate)= 1.59999996E-04
Mean S for m selected sites (corrected for mutation rate)= 9.08000022E-02
Var(Ss)= 9.08000022E-02 Cov(S,k)= 1.59999996E-04
Uncorrected estimate of mean D for selected sites= -0.248086691

Theta-w for selected sites 1.19298376E-04 Delta-theta-w= 0.986588240

Statistics for mixture of m neutral and selected sites
Mean k for mixture= 0.270088017
Mean S for mixture= 2.36287522
d= -4.03604209E-02 f(S)= 6.93297908E-02

1st and 2nd derivs wrt Sn= -0.557177365 0.195830151
1st and 2nd derivs wrt Ss= -0.376163989 5.13988771E-02
Mixed 2nd derivative= 0.165558636
1st approx. correction term for mean D= -2.32785717E-02
2nd approx. term for mean D= -2.04802640E-02
Estimate of theoretical D= -0.153283611
Corrected estimate of mean D= -0.197042450

Theta-w= 3.10448441E-03 Delta-theta-w= 0.130006850
Ratio of mixture to neutral D= 0.681258619

Uncorrected mean frequency of deleterious alleles= 1.60000002E-06

Mean k for m selected sites (corrected for mutation rate)= 3.19999992E-04
Mean S for m selected sites (corrected for mutation rate)= 0.181600004
Var(Ss)= 0.181600004 Cov(S,k)= 3.19999992E-04
Uncorrected estimate of mean D for selected sites= -0.349566966

Theta-w for selected sites 2.38596753E-04 Delta-theta-w= 0.986588240

Statistics for mixture of m neutral and selected sites
Mean k for mixture= 0.270176023
Mean S for mixture= 2.41281509
d= -4.68337834E-02 f(S)= 7.10365474E-02

1st and 2nd derivs wrt Sn= -0.537217915 0.183134183
1st and 2nd derivs wrt Ss= -0.367974460 5.00563122E-02
Mixed 2nd derivative= 0.158063039
1st approx. correction term for mean D= -2.00284123E-02
2nd approx. term for mean D= -2.23350096E-02
Estimate of theoretical D= -0.175718844
Corrected estimate of mean D= -0.218082264

Theta-w= 3.17009818E-03 Delta-theta-w= 0.147736073
Ratio of mixture to neutral D= 0.780970514

Uncorrected mean frequency of deleterious alleles= 3.20000004E-06

Mean k for m selected sites (corrected for mutation rate)=   6.39999984E-04
Mean S for m selected sites (corrected for mutation rate)=   0.363200009
Var(Ss)=   0.363200009   Cov(S,k)=   6.39999984E-04
Uncorrected estimate of mean D for selected sites= -0.490799010

Theta-w for selected sites   4.77193506E-04 Delta-theta-w=   0.986588240

Statistics for mixture of m neutral and selected sites
Mean k for mixture=   0.270352006
Mean S for mixture=   2.51269507
d=   -5.97806275E-02 f(S)=   7.44800493E-02

1st and 2nd derivs wrt Sn= -0.500395417   0.160643920
1st and 2nd derivs wrt Ss= -0.352598935   4.75684814E-02
Mixed 2nd derivative=   0.144456014
1st approx. correction term for mean D=   -1.39630875E-02
2nd approx. term for mean D=   -2.52862573E-02
Estimate of theoretical D= -0.219048604
Corrected estimate of mean D= -0.258297950

Theta-w=   3.30132642E-03 Delta-theta-w=   0.181080639
Ratio of mixture to neutral D=   0.973546684

Uncorrected mean frequency of deleterious alleles=   6.40000007E-06

Mean k for m selected sites (corrected for mutation rate)=   1.27999997E-03
Mean S for m selected sites (corrected for mutation rate)=   0.726400018
Var(Ss)=   0.726400018   Cov(S,k)=   1.27999997E-03
Uncorrected estimate of mean D for selected sites= -0.684334457

Theta-w for selected sites   9.54387011E-04 Delta-theta-w=   0.986588240

Statistics for mixture of m neutral and selected sites
Mean k for mixture=   0.270704001
Mean S for mixture=   2.71245503
d=   -8.56742561E-02 f(S)=   8.14870000E-02

1st and 2nd derivs wrt Sn= -0.437261045   0.124971546
1st and 2nd derivs wrt Ss= -0.325320333   4.32532504E-02
Mixed 2nd derivative=   0.121819682
1st approx. correction term for mean D=   -3.33206169E-03
2nd approx. term for mean D=   -2.89618578E-02
Estimate of theoretical D= -0.300127774
Corrected estimate of mean D= -0.332421690

Theta-w=   3.56378290E-03 Delta-theta-w=   0.240402579
Ratio of mixture to neutral D=   1.33389759

Uncorrected mean frequency of deleterious alleles=   1.28000001E-05

Mean k for m selected sites (corrected for mutation rate)=   2.55999994E-03
Mean S for m selected sites (corrected for mutation rate)=   1.45280004
Var(Ss)=   1.45280004   Cov(S,k)=   2.55999994E-03
Uncorrected estimate of mean D for selected sites= -0.941850662

Theta-w for selected sites   1.90877402E-03 Delta-theta-w=   0.986588240

Statistics for mixture of m neutral and selected sites
Mean k for mixture= 0.271408021
Mean S for mixture= 3.11197519
d= -0.137461543 f(S)= 9.59806815E-02

1st and 2nd derivs wrt Sn= -0.342056274 7.84196258E-02
1st and 2nd derivs wrt Ss= -0.281411707 3.65393087E-02
Mixed 2nd derivative= 8.94649327E-02
1st approx. correction term for mean D= 1.33612622E-02
2nd approx. term for mean D= -3.14523503E-02
Estimate of theoretical D= -0.443699896
Corrected estimate of mean D= -0.461790979

Theta-w= 4.08869563E-03 Delta-theta-w= 0.336198986
Ratio of mixture to neutral D= 1.97199416

Uncorrected mean frequency of deleterious alleles= 2.56000003E-05

Mean k for m selected sites (corrected for mutation rate)= 5.11999987E-03
Mean S for m selected sites (corrected for mutation rate)= 2.90560007
Var(Ss)= 2.90560007 Cov(S,k)= 5.11999987E-03
Uncorrected estimate of mean D for selected sites= -1.26665974

Theta-w for selected sites 3.81754804E-03 Delta-theta-w= 0.986588240

Statistics for mixture of m neutral and selected sites
Mean k for mixture= 0.272816002
Mean S for mixture= 3.91101503
d= -0.241036117 f(S)= 0.126887113

1st and 2nd derivs wrt Sn= -0.225033417 3.41653824E-02
1st and 2nd derivs wrt Ss= -0.220561132 2.76023503E-02
Mixed 2nd derivative= 5.30402996E-02
1st approx. correction term for mean D= 3.53037976E-02
2nd approx. term for mean D= -3.09063233E-02
Estimate of theoretical D= -0.676664472
Corrected estimate of mean D= -0.672267020

Theta-w= 5.13852155E-03 Delta-theta-w= 0.469076872
Ratio of mixture to neutral D= 3.00738931

Uncorrected mean frequency of deleterious alleles= 5.12000006E-05

Mean k for m selected sites (corrected for mutation rate)= 1.02399997E-02
Mean S for m selected sites (corrected for mutation rate)= 5.81120014
Var(Ss)= 5.81120014 Cov(S,k)= 1.02399997E-02
Uncorrected estimate of mean D for selected sites= -1.64118016

Theta-w for selected sites 7.63509609E-03 Delta-theta-w= 0.986588240

Statistics for mixture of m neutral and selected sites
Mean k for mixture= 0.275632024
Mean S for mixture= 5.50909519
d= -0.448185265 f(S)= 0.196376339

1st and 2nd derivs wrt Sn= -0.116879821 7.79919978E-03
1st and 2nd derivs wrt Ss= -0.151355505 1.77905709E-02
Mixed 2nd derivative= 2.35181749E-02
1st approx. correction term for mean D= 5.73123470E-02
2nd approx. term for mean D= -3.21835577E-02
Estimate of theoretical D= -1.01137686
Corrected estimate of mean D= -0.986248016

Theta-w= 7.23817293E-03 Delta-theta-w= 0.619196653
Ratio of mixture to neutral D= 4.49499607

Uncorrected mean frequency of deleterious alleles= 1.02400001E-04

Mean k for m selected sites (corrected for mutation rate)= 2.04799995E-02
Mean S for m selected sites (corrected for mutation rate)= 11.6224003
Var(Ss)= 11.6224003 Cov(S,k)= 2.04799995E-02
Uncorrected estimate of mean D for selected sites= -2.01921654

Theta-w for selected sites 1.52701922E-02 Delta-theta-w= 0.986588240

Statistics for mixture of m neutral and selected sites
Mean k for mixture= 0.281264007
Mean S for mixture= 8.70525551
d= -0.862483561 f(S)= 0.366060108

1st and 2nd derivs wrt Sn= -4.59245257E-02 -2.43322225E-04
1st and 2nd derivs wrt Ss= -8.83885548E-02 9.20421164E-03
Mixed 2nd derivative= 7.36780511E-03
1st approx. correction term for mean D= 7.11190477E-02
2nd approx. term for mean D= -4.55908142E-02
Estimate of theoretical D= -1.42552435
Corrected estimate of mean D= -1.39999604

Theta-w= 1.14374766E-02 Delta-theta-w= 0.754085600
Ratio of mixture to neutral D= 6.33564615

Observed Tajima D for neutral sites ≈ 0.00000000
No need to use quadratic equation for Sn: population is at or near equilibrium

Neutral diversity= 3.00000003E-03

f(S)= 6.66325763E-02 derivative= 3.37574258E-02
g(S)= 3.87397385 derivative= -0.981317163
2nd derivative of g(S)= 0.629230559
3rd derivative of g(S)= -0.678907394

Equilibrium Var(S)= 2.43131542 Cov(S,k)= 0.345079303
1st approx. correction term for mean D= -2.51598135E-02
2nd approx. correction term for mean D= 0.00000000

Uncorrected mean frequency of deleterious alleles= 1.00000001E-07

Mean k for m selected sites (corrected for mutation rate)= 1.99999995E-05
Mean S for m selected sites (corrected for mutation rate)= 1.13500003E-02
Var(Ss)= 1.13500003E-02 Cov(S,k)= 1.99999995E-05
Uncorrected estimate of mean D for selected sites= -8.79949182E-02

Theta-w for selected sites 1.49122970E-05 Delta-theta-w= 0.986588240

Statistics for mixture of m neutral and selected sites
Mean k for mixture= 0.135011002
Mean S for mixture= 1.03375030
d= -8.09177756E-04 f(S)= 2.75783017E-02

1st and 2nd derivs wrt Sn= -1.65844297 1.17337859
1st and 2nd derivs wrt Ss= -1.48024011 0.850969672
Mixed 2nd derivative= 1.22303176
1st approx. correction term for mean D= -1.79364569E-02
2nd approx. term for mean D= -1.15814060E-03
Estimate of theoretical D= -4.87259356E-03
Corrected estimate of mean D= -2.39671916E-02

Theta-w= 1.35820184E-03 Delta-theta-w= 5.95778227E-03
Ratio of mixture to neutral D= 0.193665728

Uncorrected mean frequency of deleterious alleles= 2.00000002E-07

Mean k for m selected sites (corrected for mutation rate)= 3.99999990E-05
Mean S for m selected sites (corrected for mutation rate)= 2.27000006E-02
Var(Ss)= 2.27000006E-02 Cov(S,k)= 3.99999990E-05
Uncorrected estimate of mean D for selected sites= -0.124386184

Theta-w for selected sites 2.98245941E-05 Delta-theta-w= 0.986588240

Statistics for mixture of m neutral and selected sites
Mean k for mixture= 0.135021999
Mean S for mixture= 1.03999281
d= -1.61837041E-03 f(S)= 2.77578477E-02

1st and 2nd derivs wrt Sn= -1.64237809 1.15323496
1st and 2nd derivs wrt Ss= -1.46860623 0.839701355
Mixed 2nd derivative= 1.20557070
1st approx. correction term for mean D= -1.65721625E-02
2nd approx. term for mean D= -2.28428049E-03
Estimate of theoretical D= -9.71370842E-03
Corrected estimate of mean D= -2.85701528E-02

Theta-w= 1.36640365E-03 Delta-theta-w= 1.18439794E-02
Ratio of mixture to neutral D= 0.386080295

Uncorrected mean frequency of deleterious alleles= 4.00000005E-07

Mean k for m selected sites (corrected for mutation rate)= 7.99999980E-05
Mean S for m selected sites (corrected for mutation rate)= 4.54000011E-02
Var(Ss)= 4.54000011E-02 Cov(S,k)= 7.99999980E-05
Uncorrected estimate of mean D for selected sites= -0.175746575

Theta-w for selected sites 5.96491882E-05 Delta-theta-w= 0.986588240

Statistics for mixture of m neutral and selected sites
Mean k for mixture= 0.135044008
Mean S for mixture= 1.05247784
d= -3.23671103E-03 f(S)= 2.81174108E-02

1st and 2nd derivs wrt Sn= -1.61097527 1.11427331
1st and 2nd derivs wrt Ss= -1.44583237 0.817828715
Mixed 2nd derivative= 1.17169797
1st approx. correction term for mean D= -1.39068421E-02
2nd approx. term for mean D= -4.44445619E-03
Estimate of theoretical D= -1.93026196E-02
Corrected estimate of mean D= -3.76539193E-02

Theta-w= 1.38280727E-03 Delta-theta-w= 2.34068632E-02
Ratio of mixture to neutral D= 0.767200410

Uncorrected mean frequency of deleterious alleles= 8.00000009E-07

Mean k for m selected sites (corrected for mutation rate)= 1.59999996E-04
Mean S for m selected sites (corrected for mutation rate)= 9.08000022E-02
Var(Ss)= 9.08000022E-02 Cov(S,k)= 1.59999996E-04
Uncorrected estimate of mean D for selected sites= -0.248086691

Theta-w for selected sites 1.19298376E-04 Delta-theta-w= 0.986588240

Statistics for mixture of m neutral and selected sites
Mean k for mixture= 0.135088012
Mean S for mixture= 1.07744777
d= -6.47340715E-03 f(S)= 2.88384035E-02

1st and 2nd derivs wrt Sn= -1.55094004 1.04132509
1st and 2nd derivs wrt Ss= -1.40216851 0.776583791
Mixed 2nd derivative= 1.10790384
1st approx. correction term for mean D= -8.81710835E-03
2nd approx. term for mean D= -8.42288509E-03
Estimate of theoretical D= -3.81195098E-02
Corrected estimate of mean D= -5.53595014E-02

Theta-w= 1.41561416E-03 Delta-theta-w= 4.57285643E-02
Ratio of mixture to neutral D= 1.51509511

Uncorrected mean frequency of deleterious alleles= 1.60000002E-06

Mean k for m selected sites (corrected for mutation rate)= 3.19999992E-04
Mean S for m selected sites (corrected for mutation rate)= 0.181600004
Var(Ss)= 0.181600004 Cov(S,k)= 3.19999992E-04
Uncorrected estimate of mean D for selected sites= -0.349566966

Theta-w for selected sites 2.38596753E-04 Delta-theta-w= 0.986588240

Statistics for mixture of m neutral and selected sites
Mean k for mixture= 0.135176003
Mean S for mixture= 1.12738776
d= -1.29468292E-02 f(S)= 3.02878916E-02

1st and 2nd derivs wrt Sn= -1.44094789 0.912987351
1st and 2nd derivs wrt Ss= -1.32171214 0.702987134
Mixed 2nd derivative= 0.994353890
1st approx. correction term for mean D= 4.87081707E-04
2nd approx. term for mean D= -1.51958400E-02

Estimate of theoretical D= -7.43924603E-02
Corrected estimate of mean D= -8.91012177E-02

Theta-w= 1.48122828E-03 Delta-theta-w= 8.74059796E-02
Ratio of mixture to neutral D= 2.95679688

Uncorrected mean frequency of deleterious alleles= 3.20000004E-06

Mean k for m selected sites (corrected for mutation rate)= 6.39999984E-04
Mean S for m selected sites (corrected for mutation rate)= 0.363200009
Var(Ss)= 0.363200009 Cov(S,k)= 6.39999984E-04
Uncorrected estimate of mean D for selected sites= -0.490799010

Theta-w for selected sites 4.77193506E-04 Delta-theta-w= 0.986588240

Statistics for mixture of m neutral and selected sites
Mean k for mixture= 0.135352001
Mean S for mixture= 1.22726774
d= -2.58936435E-02 f(S)= 3.32168527E-02

1st and 2nd derivs wrt Sn= -1.25462556 0.711704314
1st and 2nd derivs wrt Ss= -1.18386793 0.584264398
Mixed 2nd derivative= 0.812115550
1st approx. correction term for mean D= 1.61794517E-02
2nd approx. term for mean D= -2.51502693E-02
Estimate of theoretical D= -0.142073780
Corrected estimate of mean D= -0.151044592

Theta-w= 1.61245652E-03 Delta-theta-w= 0.160585046
Ratio of mixture to neutral D= 5.64685345

Uncorrected mean frequency of deleterious alleles= 6.40000007E-06

Mean k for m selected sites (corrected for mutation rate)= 1.27999997E-03
Mean S for m selected sites (corrected for mutation rate)= 0.726400018
Var(Ss)= 0.726400018 Cov(S,k)= 1.27999997E-03
Uncorrected estimate of mean D for selected sites= -0.684334457

Theta-w for selected sites 9.54387011E-04 Delta-theta-w= 0.986588240

Statistics for mixture of m neutral and selected sites
Mean k for mixture= 0.135704011
Mean S for mixture= 1.42702782
d= -5.17872870E-02 f(S)= 3.91947255E-02

1st and 2nd derivs wrt Sn= -0.978837013 0.452849030
1st and 2nd derivs wrt Ss= -0.975219250 0.422826260
Mixed 2nd derivative= 0.566953897
1st approx. correction term for mean D= 3.92106585E-02
2nd approx. term for mean D= -3.64623852E-02
Estimate of theoretical D= -0.261582911
Corrected estimate of mean D= -0.258834630

Theta-w= 1.87491300E-03 Delta-theta-w= 0.276211619
Ratio of mixture to neutral D= 10.3968544

Uncorrected mean frequency of deleterious alleles= 1.28000001E-05

Mean k for m selected sites (corrected for mutation rate)= 2.55999994E-03
Mean S for m selected sites (corrected for mutation rate)= 1.45280004
Var(Ss)= 1.45280004 Cov(S,k)= 2.55999994E-03
Uncorrected estimate of mean D for selected sites= -0.941850662

Theta-w for selected sites 1.90877402E-03 Delta-theta-w= 0.986588240

Statistics for mixture of m neutral and selected sites
Mean k for mixture= 0.136408001
Mean S for mixture= 1.82654786
d= -0.103574589 f(S)= 5.16302325E-02

1st and 2nd derivs wrt Sn= -0.647434354 0.208872035
1st and 2nd derivs wrt Ss= -0.713282824 0.252869487
Mixed 2nd derivative= 0.314797133
1st approx. correction term for mean D= 6.64082170E-02
2nd approx. term for mean D= -4.53243703E-02
Estimate of theoretical D= -0.455828190
Corrected estimate of mean D= -0.434744358

Theta-w= 2.39982596E-03 Delta-theta-w= 0.431592107
Ratio of mixture to neutral D= 18.1173115

Uncorrected mean frequency of deleterious alleles= 2.56000003E-05

Mean k for m selected sites (corrected for mutation rate)= 5.11999987E-03
Mean S for m selected sites (corrected for mutation rate)= 2.90560007
Var(Ss)= 2.90560007 Cov(S,k)= 5.11999987E-03
Uncorrected estimate of mean D for selected sites= -1.26665974

Theta-w for selected sites 3.81754804E-03 Delta-theta-w= 0.986588240

Statistics for mixture of m neutral and selected sites
Mean k for mixture= 0.137816012
Mean S for mixture= 2.62558794
d= -0.207149148 f(S)= 7.84203261E-02

1st and 2nd derivs wrt Sn= -0.345866799 5.94355240E-02
1st and 2nd derivs wrt Ss= -0.453954428 0.122938812
Mixed 2nd derivative= 0.131903619
1st approx. correction term for mean D= 9.00458470E-02
2nd approx. term for mean D= -5.19651771E-02
Estimate of theoretical D= -0.739722490
Corrected estimate of mean D= -0.701641798

Theta-w= 3.44965165E-03 Delta-theta-w= 0.600492954
Ratio of mixture to neutral D= 29.4009533

Uncorrected mean frequency of deleterious alleles= 5.12000006E-05

Mean k for m selected sites (corrected for mutation rate)= 1.02399997E-02
Mean S for m selected sites (corrected for mutation rate)= 5.81120014
Var(Ss)= 5.81120014 Cov(S,k)= 1.02399997E-02

Uncorrected estimate of mean D for selected sites=   -1.64118016

Theta-w for selected sites   7.63509609E-03 Delta-theta-w=   0.986588240

Statistics for mixture of m neutral and selected sites
Mean k for mixture=   0.140632004
Mean S for mixture=   4.22366810
d= -0.414298326   f(S)=   0.139676854

1st and 2nd derivs wrt Sn= -0.145500734   6.46240450E-03
1st and 2nd derivs wrt Ss= -0.252316296   5.02669886E-02
Mixed 2nd derivative=   4.11617570E-02
1st approx. correction term for mean D=   0.102855496
2nd approx. term for mean D=   -6.37654215E-02
Estimate of theoretical D=   -1.10853899
Corrected estimate of mean D=   -1.06944895

Theta-w=   5.54930326E-03 Delta-theta-w=   0.746577144
Ratio of mixture to neutral D=   44.0599060

Uncorrected mean frequency of deleterious alleles=   1.02400001E-04

Mean k for m selected sites (corrected for mutation rate)=   2.04799995E-02
Mean S for m selected sites (corrected for mutation rate)=   11.6224003
Var(Ss)=   11.6224003   Cov(S,k)=   2.04799995E-02
Uncorrected estimate of mean D for selected sites=   -2.01921654

Theta-w for selected sites   1.52701922E-02 Delta-theta-w=   0.986588240

Statistics for mixture of m neutral and selected sites
Mean k for mixture=   0.146264002
Mean S for mixture=   7.41982794
d= -0.828596532   f(S)=   0.292895228

1st and 2nd derivs wrt Sn=   -4.79163118E-02 -1.96092646E-03
1st and 2nd derivs wrt Ss= -0.123496927   1.78094301E-02
Mixed 2nd derivative=   9.60764568E-03
1st approx. correction term for mean D=   0.101776302
2nd approx. term for mean D=   -8.37796852E-02
Estimate of theoretical D=   -1.53104150
Corrected estimate of mean D=   -1.51304495

Theta-w=   9.74860601E-03 Delta-theta-w=   0.849964142
Ratio of mixture to neutral D=   60.8526573

Observed Tajima D for neutral sites ≈ 0.00000000
Neutral diversity=   6.00000005E-03
No need to use quadratic equation for Sn: population is at or near equilibrium

Equilibrium neutral values of k and S for m neutral sites
kn=   0.600000024   Sn=   4.56670141

f(S)=   0.154160082   derivative=   4.29084152E-02
g(S)=   2.54691267   derivative= -0.354449689
2nd derivative of g(S)=   0.114878483
3rd derivative of g(S)=   -6.15079738E-02

Equilibrium Var(S)= 5.15856075 Cov(S,k)= 0.780317187
1st approx. correction term for mean D= -3.63506414E-02
2nd approx. correction term for mean D= 0.00000000
Population is at or near equilibrium
Uncorrected mean frequency of deleterious alleles= 1.00000001E-07

Mean k for m selected sites (corrected for mutation rate)= 1.99999995E-05
Mean S for m selected sites (corrected for mutation rate)= 1.13500003E-02
Var(Ss)= 1.13500003E-02 Cov(S,k)= 1.99999995E-05
Uncorrected estimate of mean D for selected sites= -8.79949182E-02

Theta-w for selected sites 1.49122970E-05 Delta-theta-w= 0.986588240

Statistics for mixture of m neutral and selected sites
Mean k for mixture= 0.270011008
Mean S for mixture= 2.06125808
d= -8.09192657E-04 f(S)= 5.92341423E-02

1st and 2nd derivs wrt Sn= -0.669679284 0.264898032
1st and 2nd derivs wrt Ss= -0.470246226 8.50087032E-02
Mixed 2nd derivative= 0.229932025
1st approx. correction term for mean D= -3.05250660E-02
2nd approx. term for mean D= -5.53268299E-04
Estimate of theoretical D= -3.32480273E-03
Corrected estimate of mean D= -3.44031379E-02

Theta-w= 2.70820200E-03 Delta-theta-w= 2.98792124E-03
Ratio of mixture to neutral D= 9.14647579E-02

Uncorrected mean frequency of deleterious alleles= 2.00000002E-07

Mean k for m selected sites (corrected for mutation rate)= 3.99999990E-05
Mean S for m selected sites (corrected for mutation rate)= 2.27000006E-02
Var(Ss)= 2.27000006E-02 Cov(S,k)= 3.99999990E-05
Uncorrected estimate of mean D for selected sites= -0.124386184

Theta-w for selected sites 2.98245941E-05 Delta-theta-w= 0.986588240

Statistics for mixture of m neutral and selected sites
Mean k for mixture= 0.270022005
Mean S for mixture= 2.06750059
d= -1.61835551E-03 f(S)= 5.94393946E-02

1st and 2nd derivs wrt Sn= -0.666213453 0.262402236
1st and 2nd derivs wrt Ss= -0.468675762 8.46053585E-02
Mixed 2nd derivative= 0.228372782
1st approx. correction term for mean D= -2.99871489E-02
2nd approx. term for mean D= -1.09687157E-03
Estimate of theoretical D= -6.63799234E-03
Corrected estimate of mean D= -3.77220102E-02

Theta-w= 2.71640369E-03 Delta-theta-w= 5.95778227E-03
Ratio of mixture to neutral D= 0.182610050

Uncorrected mean frequency of deleterious alleles= 4.00000005E-07

Mean k for m selected sites (corrected for mutation rate)= 7.99999980E-05
Mean S for m selected sites (corrected for mutation rate)= 4.54000011E-02
Var(Ss)= 4.54000011E-02 Cov(S,k)= 7.99999980E-05
Uncorrected estimate of mean D for selected sites= -0.175746575

Theta-w for selected sites 5.96491882E-05 Delta-theta-w= 0.986588240

Statistics for mixture of m neutral and selected sites
Mean k for mixture= 0.270043999
Mean S for mixture= 2.07998562
d= -3.23674083E-03 f(S)= 5.98503686E-02

1st and 2nd derivs wrt Sn= -0.659363270 0.257497966
1st and 2nd derivs wrt Ss= -0.465565383 8.38091150E-02
Mixed 2nd derivative= 0.225299433
1st approx. correction term for mean D= -2.89225969E-02
2nd approx. term for mean D= -2.15586997E-03
Estimate of theoretical D= -1.32304467E-02
Corrected estimate of mean D= -4.43089157E-02

Theta-w= 2.73280730E-03 Delta-theta-w= 1.18439794E-02
Ratio of mixture to neutral D= 0.363967359

Uncorrected mean frequency of deleterious alleles= 8.00000009E-07

Mean k for m selected sites (corrected for mutation rate)= 1.59999996E-04
Mean S for m selected sites (corrected for mutation rate)= 9.08000022E-02
Var(Ss)= 9.08000022E-02 Cov(S,k)= 1.59999996E-04
Uncorrected estimate of mean D for selected sites= -0.248086691

Theta-w for selected sites 1.19298376E-04 Delta-theta-w= 0.986588240

Statistics for mixture of m neutral and selected sites
Mean k for mixture= 0.270088017
Mean S for mixture= 2.10495567
d= -6.47342205E-03 f(S)= 6.06741905E-02

1st and 2nd derivs wrt Sn= -0.645979941 0.248026937
1st and 2nd derivs wrt Ss= -0.459463537 8.22570324E-02
Mixed 2nd derivative= 0.219327614
1st approx. correction term for mean D= -2.68375613E-02
2nd approx. term for mean D= -4.16542357E-03
Estimate of theoretical D= -2.62803975E-02
Corrected estimate of mean D= -5.72833829E-02

Theta-w= 2.76561454E-03 Delta-theta-w= 2.34068632E-02
Ratio of mixture to neutral D= 0.722969294

Uncorrected mean frequency of deleterious alleles= 1.60000002E-06

Mean k for m selected sites (corrected for mutation rate)= 3.19999992E-04
Mean S for m selected sites (corrected for mutation rate)= 0.181600004
Var(Ss)= 0.181600004 Cov(S,k)= 3.19999992E-04
Uncorrected estimate of mean D for selected sites= -0.349566966

Theta-w for selected sites   2.38596753E-04 Delta-theta-w=   0.986588240

Statistics for mixture of m neutral and selected sites
Mean k for mixture=   0.270176023
Mean S for mixture=   2.15489554
d=   -1.29468143E-02 f(S)=   6.23293258E-02

1st and 2nd derivs wrt Sn= -0.620420814   0.230348915
1st and 2nd derivs wrt Ss= -0.447715163   7.93057680E-02
Mixed 2nd derivative=   0.208044216
1st approx. correction term for mean D=   -2.28358209E-02
2nd approx. term for mean D=   -7.78537802E-03
Estimate of theoretical D=   -5.18581122E-02
Corrected estimate of mean D=   -8.24793130E-02

Theta-w=   2.83122831E-03 Delta-theta-w=   4.57285643E-02
Ratio of mixture to neutral D=   1.42660785

Uncorrected mean frequency of deleterious alleles=   3.20000004E-06

Mean k for m selected sites (corrected for mutation rate)=   6.39999984E-04
Mean S for m selected sites (corrected for mutation rate)=   0.363200009
Var(Ss)=   0.363200009   Cov(S,k)=   6.39999984E-04
Uncorrected estimate of mean D for selected sites= -0.490799010

Theta-w for selected sites   4.77193506E-04 Delta-theta-w=   0.986588240

Statistics for mixture of m neutral and selected sites
Mean k for mixture=   0.270352006
Mean S for mixture=   2.25477552
d=   -2.58936584E-02 f(S)=   6.56695887E-02

1st and 2nd derivs wrt Sn= -0.573691666   0.199439704
1st and 2nd derivs wrt Ss= -0.425887108   7.39506111E-02
Mixed 2nd derivative=   0.187834963
1st approx. correction term for mean D=   -1.54479975E-02
2nd approx. term for mean D=   -1.36677166E-02
Estimate of theoretical D= -0.101044171
Corrected estimate of mean D= -0.130159885

Theta-w=   2.96245655E-03 Delta-theta-w=   8.74059796E-02
Ratio of mixture to neutral D=   2.77970815

Uncorrected mean frequency of deleterious alleles=   6.40000007E-06

Mean k for m selected sites (corrected for mutation rate)=   1.27999997E-03
Mean S for m selected sites (corrected for mutation rate)=   0.726400018
Var(Ss)=   0.726400018   Cov(S,k)=   1.27999997E-03
Uncorrected estimate of mean D for selected sites= -0.684334457

Theta-w for selected sites   9.54387011E-04 Delta-theta-w=   0.986588240

Statistics for mixture of m neutral and selected sites
Mean k for mixture=   0.270704001
Mean S for mixture=   2.45453548
d=   -5.17872870E-02 f(S)=   7.24700540E-02

1st and 2nd derivs wrt Sn= -0.494865447 0.151555449
1st and 2nd derivs wrt Ss= -0.387887537 6.50176108E-02
Mixed 2nd derivative= 0.155022055
1st approx. correction term for mean D= -2.75030918E-03
2nd approx. term for mean D= -2.14667842E-02
Estimate of theoretical D= -0.192372888
Corrected estimate of mean D= -0.216589987

Theta-w= 3.22491303E-03 Delta-theta-w= 0.160585046
Ratio of mixture to neutral D= 5.29214573

Uncorrected mean frequency of deleterious alleles= 1.28000001E-05

Mean k for m selected sites (corrected for mutation rate)= 2.55999994E-03
Mean S for m selected sites (corrected for mutation rate)= 1.45280004
Var(Ss)= 1.45280004 Cov(S,k)= 2.55999994E-03
Uncorrected estimate of mean D for selected sites= -0.941850662

Theta-w for selected sites 1.90877402E-03 Delta-theta-w= 0.986588240

Statistics for mixture of m neutral and selected sites
Mean k for mixture= 0.271408021
Mean S for mixture= 2.85405564
d= -0.103574574 f(S)= 8.65507647E-02

1st and 2nd derivs wrt Sn= -0.379156619 9.14665014E-02
1st and 2nd derivs wrt Ss= -0.328633249 5.20356074E-02
Mixed 2nd derivative= 0.109973192
1st approx. correction term for mean D= 1.65400393E-02
2nd approx. term for mean D= -2.83500608E-02
Estimate of theoretical D= -0.352060825
Corrected estimate of mean D= -0.363870829

Theta-w= 3.74982599E-03 Delta-theta-w= 0.276211619
Ratio of mixture to neutral D= 9.68513393

Uncorrected mean frequency of deleterious alleles= 2.56000003E-05

Mean k for m selected sites (corrected for mutation rate)= 5.11999987E-03
Mean S for m selected sites (corrected for mutation rate)= 2.90560007
Var(Ss)= 2.90560007 Cov(S,k)= 5.11999987E-03
Uncorrected estimate of mean D for selected sites= -1.26665974

Theta-w for selected sites 3.81754804E-03 Delta-theta-w= 0.986588240

Statistics for mixture of m neutral and selected sites
Mean k for mixture= 0.272816002
Mean S for mixture= 3.65309572
d= -0.207149178 f(S)= 0.116631262

1st and 2nd derivs wrt Sn= -0.242383212 3.75523940E-02
1st and 2nd derivs wrt Ss= -0.250283808 3.65094170E-02
Mixed 2nd derivative= 6.21532723E-02
1st approx. correction term for mean D= 4.06601354E-02
2nd approx. term for mean D= -3.10514625E-02

Estimate of theoretical D= -0.606562734
Corrected estimate of mean D= -0.596954048

Theta-w= 4.79965191E-03 Delta-theta-w= 0.431592107
Ratio of mixture to neutral D= 16.6864376

Uncorrected mean frequency of deleterious alleles= 5.12000006E-05

Mean k for m selected sites (corrected for mutation rate)= 1.02399997E-02
Mean S for m selected sites (corrected for mutation rate)= 5.81120014
Var(Ss)= 5.81120014 Cov(S,k)= 1.02399997E-02
Uncorrected estimate of mean D for selected sites= -1.64118016

Theta-w for selected sites 7.63509609E-03 Delta-theta-w= 0.986588240

Statistics for mixture of m neutral and selected sites
Mean k for mixture= 0.275632024
Mean S for mixture= 5.25117588
d= -0.414298296 f(S)= 0.184468612

1st and 2nd derivs wrt Sn= -0.121854417 7.75084272E-03
1st and 2nd derivs wrt Ss= -0.166244864 2.17000153E-02
Mixed 2nd derivative= 2.61018928E-02
1st approx. correction term for mean D= 6.32514060E-02
2nd approx. term for mean D= -3.44046429E-02
Estimate of theoretical D= -0.964610279
Corrected estimate of mean D= -0.935763478

Theta-w= 6.89930329E-03 Delta-theta-w= 0.600492954
Ratio of mixture to neutral D= 26.5362663

Uncorrected mean frequency of deleterious alleles= 1.02400001E-04

Mean k for m selected sites (corrected for mutation rate)= 2.04799995E-02
Mean S for m selected sites (corrected for mutation rate)= 11.6224003
Var(Ss)= 11.6224003 Cov(S,k)= 2.04799995E-02
Uncorrected estimate of mean D for selected sites= -2.01921654

Theta-w for selected sites 1.52701922E-02 Delta-theta-w= 0.986588240

Statistics for mixture of m neutral and selected sites
Mean k for mixture= 0.281264007
Mean S for mixture= 8.44733620
d= -0.828596652 f(S)= 0.350848645

1st and 2nd derivs wrt Sn= -4.64563742E-02 -5.04030846E-04
1st and 2nd derivs wrt Ss= -9.41987038E-02 1.04644680E-02
Mixed 2nd derivative= 7.77627015E-03
1st approx. correction term for mean D= 7.59087652E-02
2nd approx. term for mean D= -4.93106805E-02
Estimate of theoretical D= -1.39888906
Corrected estimate of mean D= -1.37229097

Theta-w= 1.10986065E-02 Delta-theta-w= 0.746577144
Ratio of mixture to neutral D= 38.4832001

Computer Code for Tajima's D calculations (FORTRAN 95)

```
 program Tajd4
!   program for Tajima's D with strong selection
!   several different selection strengths
    real :: D,pin,Mn,ks,kt

    CHARACTER*20 FINP
    CHARACTER*20 FOUT

    WRITE (*,*) 'Input file?'
    READ (*,*) FINP
    OPEN (2,FILE=FINP)
    WRITE (*,*) 'Output file?'
    READ (*,*) FOUT
    OPEN (1,FILE=FOUT)
    write (1,*) 'Tajima D with mixture of neutrality and strong selection'
    write (1,*) 'Different mutation rates for neutral and mixed sequences'
    write (1,*) ''

    READ (2,*)  n
    READ (2,*)  D
    READ (2,*) pin
    READ (2,*) m,pn
    READ (2,*) R
    READ (2,*) ndat

    write (1,*) 'Sample size= ',n
    write (1,*) 'Observed Tajima D for neutral sites = ',D
    write (1,*) 'Neutral diversity= ',pin
    write (1,*) 'Total number of sites in a segment= ',m
    write (1,*) 'Proportion of neutral sites in a segment= ',pn
    write (1,*) 'Ratio of mutation rates (mixed/pure neutral)= ',R
    write (1,*) 'Number of data items= ',ndat
    write (1,*) ''

    write (*,*) 'Sample size= ',n
    write (*,*) 'Observed Tajima D for neutral sites= ',D
    write (*,*) 'Neutral diversity= ',pin
    write (*,*) 'Total number of sites in a segment (m)= ',m
    write (*,*) 'Proportion of neutral sites in a segment= ',pn
    write (*,*) 'Ratio of mutation rates (mixed/pure neutral)= ',R
    write (*,*) 'Number of data items= ',ndat
    write (*,*) ''

    write (*,*) 'Continue?'
    read (*,*) icont
!   allows program to be cancelled if desired
    if(icont.gt.0) go to 100

!!!!!!!!!!!!!!!!!!!!!!!!!!!!!!!!!!!!!!!!!!!!!!!!!!!!!!!!!!!!!!!!!!!!!!!!
    a1=0
    a2=0
    n1=n-1
    do 10 i=1,n1
    ai=i
    a1=a1+1/ai
    a2=a2+1/(ai**2)
```

```
10 continue

   an=n
   an1=n-1
   b1=(an+1)/(3.0*(an-1))
   b2=2*(an*(an+1)+3)/(9.0*an*an1)
   c1=b1-(1.0/a1)
   c2=b2+(a2/a1**2)-((an+2)/(an*a1))
   e1=c1/a1
   e2=c2/(a2+a1**2)
   write (1,*) 'Coefficients needed for the Tajima D calculations'
   write (1,*) 'a1= ',a1, 'a2= ',a2
   write (1,*) 'b1= ',b1, 'b2= ',b2
   write (1,*) 'c1= ',c1, 'c2= ',c2
   write (1,*) 'e1= ',e1, 'e2= ',e2
!!!!!!!!!!!!!!!!!!!!!!!!!!!!!!!!!!!!!!!!!!!!!!!!!!!!!!!!!!!!!!!!!!!!!!!!
!  determination of S for m neutral sites
   Mn=m*pin
!  equilibrium neutral mean number of pairwise differences
   Sn=m*pin*a1
   write (1,*) 'Equilibrium neutral values of k and S for m neutral sites'
   write (1,*) 'kn= ',Mn, 'Sn= ',Sn

   S1=Sn
   do 30 i=1,5
   fS=S1*((e1-e2)+S1*e2)
   gS=1.0/sqrt(fS)
   dfS=e1-e2+2*S1*e2
   write (1,*) ''
   write (1,*) 'Iteration=',i
   write (1,*) ''
   write (1,*) 'f(S)= ',fS,' derivative= ',dfS
   dgS=0-0.5*dfS/(fS**1.5)
   write (1,*) 'g(S)= ',gS,' derivative= ',dgS
   d2gS=0.0-e2*(fS**(0-1.5))
   d2gS=d2gS+0.75*(fS**(0-2.5))*(dfS**2)
   write (1,*) '2nd derivative of g(S)= ',d2gS
   d3gS=0.0-(15/8.0)*(fS**(0-3.5))*(dfS**3)
   d3gS=d3gS+e2*dfS*4.5*(fS**(0-2.5))
   write (1,*) '3nd derivative of g(S)= ',d3gS
   write (1,*) ''

   write (*,*) 'f(S)= ',fS,' derivative= ',dfS
   write (*,*) 'g(S)= ',gS,' derivative= ',dgS
   write (*,*) '2nd derivative of g(S)= ',d2gS
   write (*,*) '3nd derivative of g(S)= ',d3gS
   varn=a1*Mn+a2*Mn**2
   covn=Mn+(0.5+1.0/an)*Mn**2
   write (1,*) ''
   write (1,*) 'Equilibrium Var(S)= ',varn,' Cov(S,k)= ',covn
   C1=(covn-varn/a1)*dgs
   D1=D-C1
   write (1,*) '1st approx. correction term for mean D= ',C1
   C11=0.5*varn*(Mn-S1/a1)*d2gs
   write (1,*) '2nd approx. correction term for mean D= ',C11
   D1=D1-C11

   if(abs(D).le.0.01) then
```

```
   S1=Sn
   write (1,*) 'Population is at or near equilibrium'
   write (1,*) 'No need to use quadratic equation for Sn'
   write (1,*) ''
   Dn=D+C1
   go to 15
   end if

   a=(1.0/a1**2)-(D1**2)*e2
   b=0.0-((D1**2)*(e1-e2)+2*Mn/a1)
   c=Mn**2
   del=sqrt((b**2)-4*a*c)
   write (1,*) 'Coefficients of quadratic for S'
   write (1,*) 'a= ',a,'b= ',b,'c=',c,' discrim= ',del
   S1=(0.0-b+del)/(2*a)
   thetw=S1/(m*a1)
   deltw=1-(a1*Mn/S1)
   write (1,*) ''
   write (1,*) 'Estimate of neutral mean S= ',S1
   write (1,*) 'Theta-w= ',thetw,' Delta-theta-w= ',deltw
   fS1=S1*((e1-e2)+S1*e2)
   fS2=sqrt(fS1)
   D2=(Mn-S1/a1)/fS2
   Dn=D2+C1+C11

   write (1,*) 'Check on D= ',Dn
   write (1,*) ''
   write (*,*) ''
   write (*,*) 'Estimate of neutral mean S= ',S1
   write (*,*) 'Theta-w= ',thetw,' Delta-theta-w= ',deltw
   write (*,*) '1st approx. correction term for mean D= ',C1
   write (*,*) 'Check on D= ',Dn
   write (*,*) '2nd approx. correction term for mean D= ',C11
30  continue

!!!!!!!!!!!!!!!!!!!!!!!!!!!!!!!!!!!!!!!!!!!!!!!!!!!!!!!!!!!!!!!!!!!!!!
!   determination of k,S and D for selected sites
15  write (1,*) 'Singletons only are assumed for selected sites'
   write (1,*) ''
   do 20 j=1,ndat
   read (2,*) qb
   ks=2*m*qb*R
   Ss=m*n*qb*R
   write (1,*) ''
   write (1,*) 'Uncorrected mean frequency of deleterious alleles= ',qb
   write (1,*) ''
   write (1,*) 'Mean k for m selected sites (corrected for mutation rate)= ',ks
   write (1,*) 'Mean S for m selected sites (corrected for mutation rate)= ',Ss

   vars=Ss
   covs=ks
   fs=Ss*(e1-e2+e2*Ss)
   D3=(ks-Ss/a1)/sqrt(fs)

   write (1,*) 'Var(Ss)= ',vars,' Cov(S,k)= ',covs
   write (1,*) 'Uncorrected estimate of mean D for selected sites= ',D3
   write (1,*) ''
   thetw=Ss/(m*a1)
```

```
   deltw=1-(a1*ks/Ss)
   write (1,*) 'Theta-w for selected sites ',thetw,' Delta-theta-w= ',deltw
   write (1,*) ''

!!!!!!!!!!!!!!!!!!!!!!!!!!!!!!!!!!!!!!!!!!!!!!!!!!!!!!!!!!!!!!!!!!!!!!!!
!  determination of k,S and D for mixture of neutral and selected sites
   kt=pn*R*Mn+(1-pn)*ks
   St=pn*R*S1+(1-pn)*Ss
!  k and S multiplied  by factor R to allow a different mutation rate for mixed sites
   ft=St*((e1-e2)+St*e2)
   gt1=ft**(0-1.5)
   dfn=pn*(e1-e2+2*S1*e2)
   dfs=(1-pn)*(e1-e2+2*Ss*e2)
   dgn=0-0.5*gt1*dfn
   dgs=0-0.5*gt1*dfs

   gt2=ft**(0-2.5)
   d2gn=0.75*gt2*(dfn**2.0)
   d2gn=d2gn-pn*gt1*e2
   d2gs=0.75*gt2*(dfs**2.0)
   d2gs=d2gs-(1-pn)*gt1*e2
   d2gns=0.75*gt2*dfn*dfs

   DU=kt-St/a1
   C4=(pn*(covn-varn/a1)*dgn)+((1-pn)*(covs-vars/a1)*dgs)
   D4=DU/sqrt(ft)
   C5=DU*0.5*(varn*d2gn+vars*d2gs)
   D5=D4+C4+C5
   write (1,*) 'Statistics for mixture of m neutral and selected sites'
   write (1,*) 'Mean k for mixture= ',kt
   write (1,*) 'Mean S for mixture= ',St
   write (1,*) 'd= ',DU,'f(S)= ',ft
   write (1,*) ''
   write (1,*)  '1st and 2nd derivs wrt Sn= ',dgn,d2gn
   write (1,*)  '1st and 2nd derivs wrt Ss= ',dgs,d2gs
   write (1,*)  'Mixed 2nd derivative= ',d2gns
   write (1,*) '1st approx. correction term for mean D= ',C4
   write (1,*) '2nd approx. term for mean D= ',C5
   write (1,*) 'Estimate of theoretical D= ',D4
   write (1,*) 'Corrected estimate of mean D= ',D5
   write (1,*) ''
   thetw=St/(m*a1)
   deltw=1-(a1*kt/St)
   write (1,*) 'Theta-w= ',thetw,' Delta-theta-w= ',deltw
   pd=D4/Dn
   write (1,*) 'Ratio of mixture to neutral D= ',pd
   write (1,*) ''
20  continue

100 end program Tajd4
```
